# Dynamic sensitivity to resource availability influences population responses to mismatches in a shorebird

**DOI:** 10.1002/ecy.3743

**Published:** 2022-06-12

**Authors:** Luke R. Wilde, Josiah E. Simmons, Rose J. Swift, Nathan R. Senner

**Affiliations:** ^1^ Department of Biological Sciences University of South Carolina Columbia South Carolina USA; ^2^ Division of Biological Sciences University of Montana Missoula Montana USA; ^3^ U.S. Geological Survey Northern Prairie Wildlife Research Center Jamestown North Dakota USA

**Keywords:** chick survival, hierarchical model, *Limosa haemastica*, phenological mismatch, resource availability

## Abstract

Climate change has caused shifts in seasonally recurring biological events leading to the temporal decoupling of consumer–resource pairs, that is, phenological mismatching. Although mismatches often affect individual fitness, they do not invariably scale up to affect populations, making it difficult to assess the risk they pose. Individual variation may contribute to this inconsistency, with changes in resource availability and consumer needs leading mismatches to have different outcomes over time. Nevertheless, most models estimate a consumer's match from a single time point, potentially obscuring when mismatches matter to populations. We analyzed how the effects of mismatches varied over time by studying precocial Hudsonian godwit (*Limosa haemastica*) chicks and their invertebrate prey from 2009 to 2019. We developed individual‐ and population‐level models to determine how age‐specific variation affects the relationship between godwits and resource availability. We found that periods with abundant resources led to higher growth and survival of godwit chicks, but also that chick survival was increasingly related to the availability of larger prey as chicks aged. At the population level, estimates of mismatches using age‐structured consumer demand explained more variation in annual godwit fledging rates than more commonly used alternatives. Our study suggests that modeling the effects of mismatches as the disrupted interaction between dynamic consumer needs and resource availability clarifies when mismatches matter to both individuals and populations.

## INTRODUCTION

Climate change is frequently linked to shifts in the timing of recurring biological events (i.e., phenology). Higher spring temperatures have led to earlier resource peaks (Pearce‐Higgins et al., 2005) and less predictable resource pulses (Visser et al., 2012). However, slower phenological response rates by upper‐trophic‐level species mean that future climatic change will likely lead to an increased decoupling of consumer–resource pairs (i.e., “mismatches”; Durant et al., 2005). Despite the theoretical risks posed by mismatches, they do not invariably lead to reduced individual fitness (Dunn et al., 2011) or negative effects for populations (Visser et al., 2012; Reed et al., 2013). These inconsistent effects may be due to among‐individual variation (Reed et al., 2013) or how the consumer–resource relationship is modeled (Visser & Gienapp, 2019). Overcoming the apparent empirical‐theoretical disconnect in phenological studies may therefore require an improved mechanistic framework to help elucidate when mismatches are likely to affect populations (Takimoto & Sato, 2020).

The match‐mismatch hypothesis defines mismatches as the asynchrony between seasonal resource availability and consumer demands leading to population level consequences (Cushing, 1990). Currently, consumers are considered mismatched when their period of greatest energetic demand (i.e., peak demand) is early or late relative to peak resource abundance. However, mismatches may not equate to asynchrony, nor does being “matched” guarantee that consumers have sufficient food (Saalfeld et al., 2019). Reduced resource availability, and not asynchrony per se, is the likely driver of the fitness consequences of mismatches to individuals on a daily basis (Samplonius et al., 2016). Still, most studies do not quantify the effects of mismatches in terms of resources (Durant et al., 2005). Moreover, a consumer's energetic demand and foraging efficiency (i.e., their ability to handle and capture prey of a particular size) changes throughout development (Schekkerman & Boele, 2009). This means that an individual's sensitivity to resource availability is not constant, but instead is likely age‐structured (Samplonius et al., 2016). Conceptualizing a mismatch as the disrupted interaction between dynamic consumer needs and resource availability, instead of as asynchrony relative to a single time point, may therefore clarify when mismatches matter to populations (Kerby et al., 2012; Yang & Rudolf, 2010).

**FIGURE 1:**
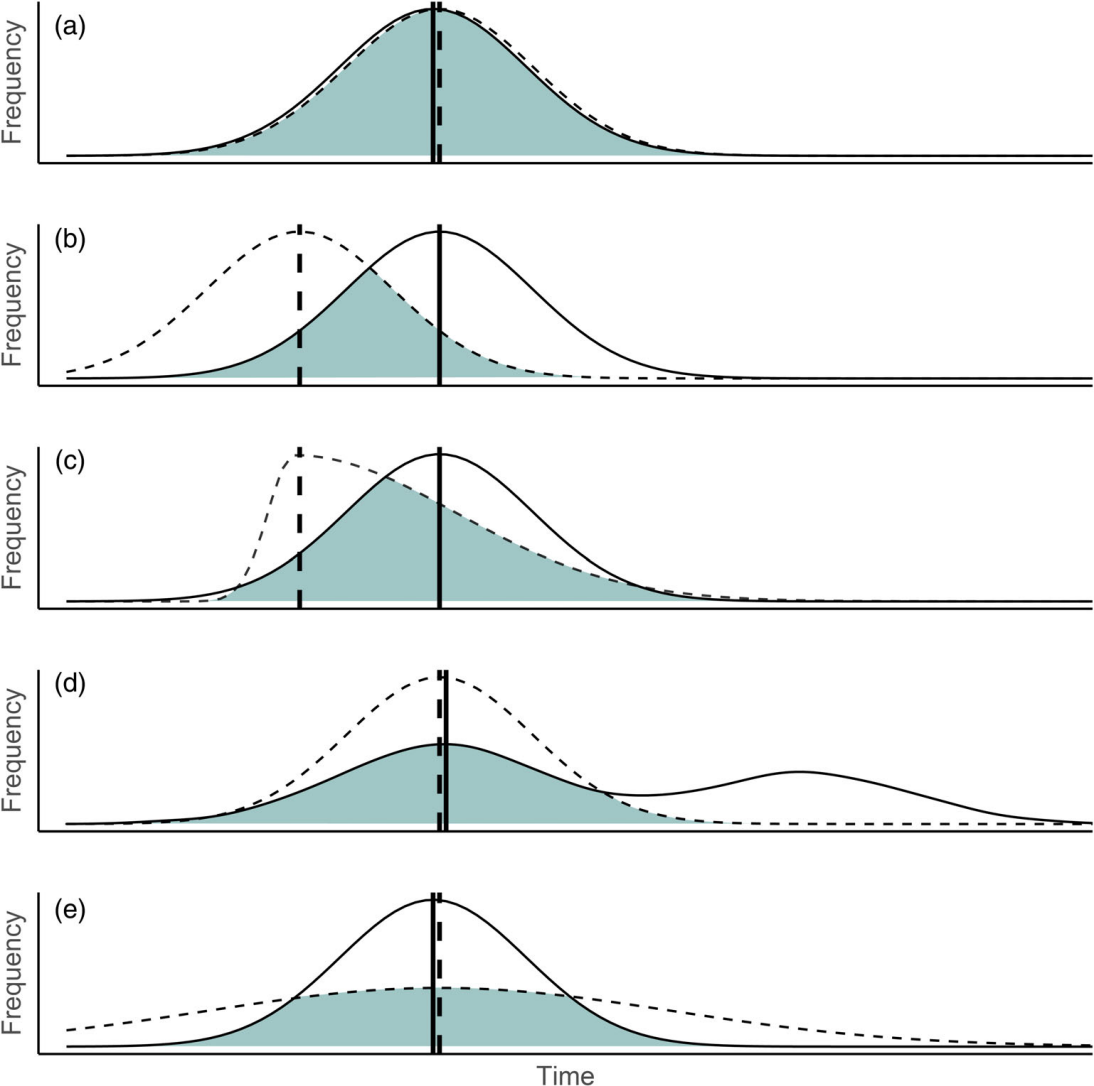
Peak dates (vertical lines) and frequency curves of consumers (solid) and resources (dashed). Difference in peak dates and overlap (shaded area) estimates are equivalent when the consumer and resource curves are symmetrical (a, b). Difference in peak date and overlap model estimates differ when either curve is skewed (c), the consumer phenology is multimodal (d), or the curves are aligned but have low overlapping area due to reduced resource abundance (e).

Incorporating consumer age structure into existing models likely requires a re‐examination of the statistical concept of mismatches (Kerby et al., 2012). Phenologies are generally modeled as frequency curves on a temporal axis (Figure 1; Visser & Gienapp, 2019), whereby a population's degree of match with their resources is estimated as the difference in peak dates (i.e., “dates” models) or proportion of overlapping area (i.e., “overlap” models). However, difference in dates models have been criticized (Lindén, 2018): while the models agree if consumer and resource curves are symmetrical (Figure 1a,b), difference in dates models can be biased when phenologies are skewed or multimodal, or in cases of reduced resource availability (Figure 1c,d,e). Because overlap models capture the magnitude and duration of the interaction between consumer demand and resource availability (Kerby et al., 2012), they may better capture the mechanisms underlying a mismatch.

**FIGURE 2:**
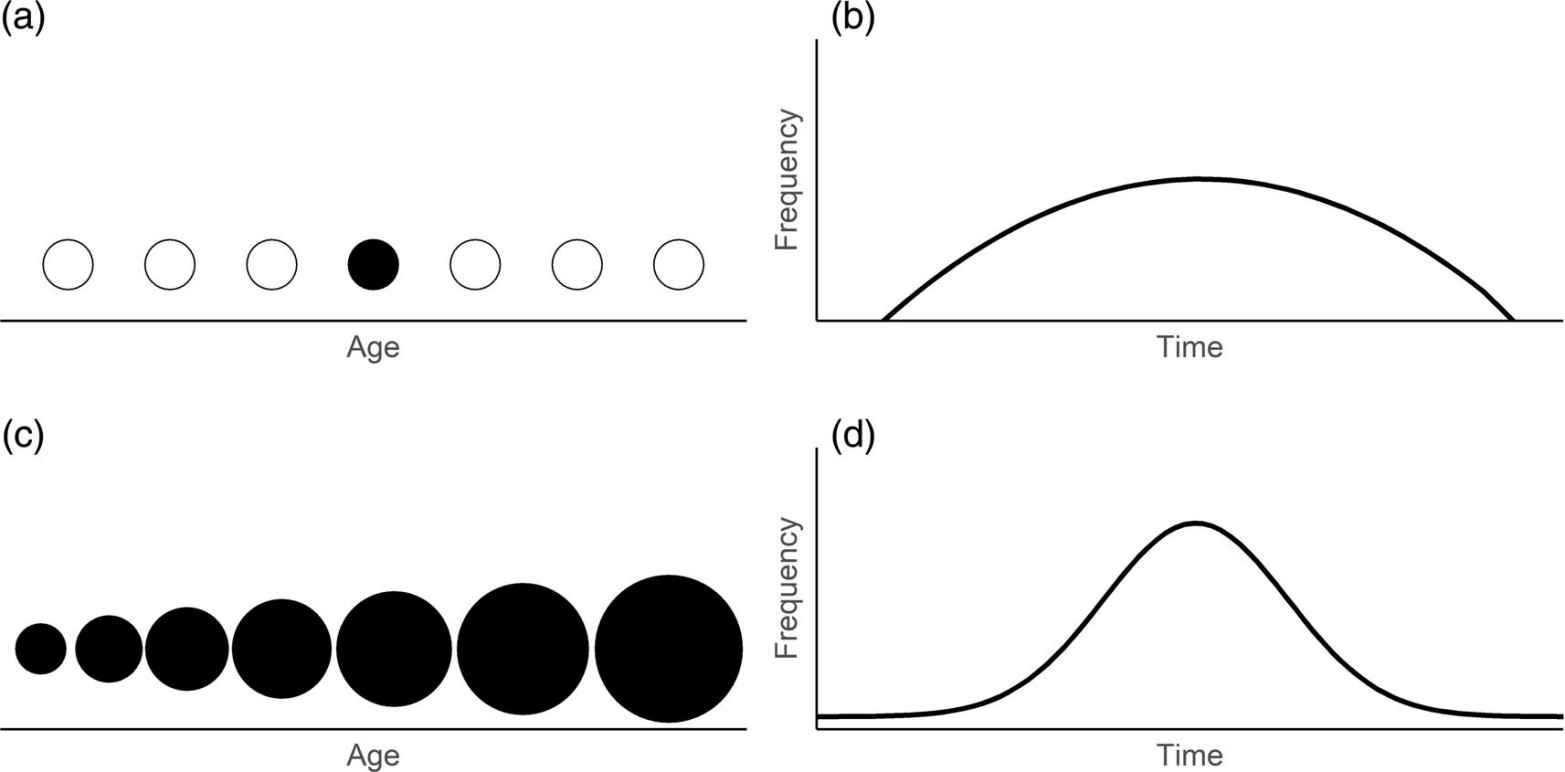
The peak demand model estimates consumer phenologies from the daily frequency of individuals at a single point in development (e.g., peak growth rate; [a]), resulting in a truncated curve (b). Since resource demand increases throughout development (c), the whole demand model includes the complete demand of all individuals continuously (d). Filled circles are time points in development. Circle size reflects hypothetical energy requirements at each timepoint.

Overlap models have also performed inconsistently (Ramakers et al., 2020). Existing overlap models estimate consumer demand from a single life‐history event or timepoint in development, such as when individual growth rates are maximized (“peak demand;” Figure 2a; Leung et al., 2018). By drawing phenologies from one timepoint, “peak demand” models ignore demand prior to or following this maxima and impose a symmetrical shape on the demand curve (Figure 2b; Kerby et al., 2012; Lindén, 2018). Because animals require increasing energy and have different foraging capabilities as they develop, their sensitivity to reduced resource availability is likely to change over time. As a result, measuring the consequences of a mismatch from a single timepoint could shroud cumulative effects (Yang & Rudolf, 2010) and mask variation in the consequences of mismatches among age‐classes (Reed et al., 2013). The growing availability of metabolic data and advances in Bayesian survival analyses may help overcome these shortcomings as they allow for direct tests of the potential age‐specific effects of mismatches. By modeling consumer demand as a function of the population age‐structure, a “whole demand” model incorporates the increasing metabolic demands of individuals as they age (Figure 2c). As a result, a whole demand curve may better quantify overlap at the demand curve's upper tail when per‐capita consumer demands are greatest and consumers are most sensitive to reduced resource availability (Figure 2d; Kerby et al., 2012).

Migratory birds provide a powerful system to examine the ways in which the effects of mismatches vary for individuals and populations. Long‐distance migrants represent some of the canonical examples of mismatches, but while many studies have identified individual‐level fitness effects resulting from mismatches, few have found corresponding population‐level consequences (Dunn et al., 2011; Visser & Both, 2005). Hudsonian godwits (*Limosa haemastica*; “godwits”) are a case in point. godwits breed in three disjunct populations spread across the Nearctic (Walker et al., 2020). Like other shorebirds (Kwon et al., 2019), godwits breeding in Alaska have kept pace with recent phenological changes in peak resource availability while those breeding in Hudson Bay have not (Senner, 2012). Despite mismatches affecting the survival of individual godwit chicks in Hudson Bay, there have been few apparent population level consequences (Senner et al., 2017). Furthermore, much of the interannual variation in the fledging rates of Alaskan godwits is not explained by predation or density‐dependent processes (Senner et al., 2017; Swift et al., 2017a, 2019; Wilde, Swift, & Senner, 2022). The observed variation in the fledging rates of Alaskan godwits may instead result from a potential correlation between early snowmelt and low annual fledging rates, suggesting that heretofore undocumented mismatches may be affecting godwits' annual reproductive output (Saalfeld et al., 2019).

Modeling mismatches along the age‐specific continuum of consumer demand may capture the effects of resource availability on consumer fitness that our previous attempts have missed. We therefore investigated how dynamic consumer needs and resource characteristics interact to influence the potential for mismatches in the Alaskan godwit population. We developed mechanistic models that integrate age‐structured consumer needs and resource availability information at the individual and population levels. We first explored how the timing, abundance, and quality of resources have changed over the course of the study. Then, we investigated the effects of invertebrate biomass and body mass on the growth and survival of godwit chicks. We hypothesized that mismatches affect individual fitness differently throughout development and predicted that chick growth and survival would improve with more abundant and high‐quality (i.e., larger) prey, but that the effect of prey body mass would increase as chicks aged (i.e., required more energy). Last, we investigated the influence mismatches have on godwit annual reproductive output. We hypothesized that mismatches are simultaneously a function of both consumer needs and resource availability. We therefore predicted the “whole demand” model would explain more of the interannual variation in godwit fledging success than alternatives. Identifying how resources interact with consumer needs will deepen our understanding of the mechanisms underlying mismatches.

## METHODS

### Study area and godwit chick monitoring

During 2009–2011, 2014–2016, and 2019, we monitored godwits on two plots, North (550 ha) and South (120 ha), near Beluga River, Alaska (61.21° N, 151.03° W; “Beluga;” Appendix S1: Figure S1). Both plots consist of ponds and black spruce stands (*Picea mariana*) dominated by dwarf shrub and graminoids surrounded by boreal forest (Swift et al., 2017a, 2017b).

Each year (early May to mid‐July: *x̄* = 78 days; Appendix S1: Table S1), we located all godwit nests in both plots using behavioral observations, past locations, and opportunistic encounters, and located, on average, 23 nests per year (range: 11–33 nests). We monitored nests every 2–3 days and captured newly hatched chicks, collecting morphometric measurements and body masses on all chicks in the brood. We uniquely marked chicks with a leg flag and U.S. Geological Survey metal band. Radios and flags together were <3% of a chick's mass at hatch and unlikely to affect their survival (Lees et al., 2019; Sharpe et al., 2009). Some nests hatched before detection (range: 0–4 year^−1^). We opportunistically captured chicks from these broods off‐nest and estimated their age from mass measurements. Because we included these chicks, we are confident that we found all broods each year given the small size of the study area and the conspicuousness of godwit broods. All procedures met the ethical standards of Cornell University (2001–0051) and the University of South Carolina (2449‐101417‐042219), Alaska Department of Fish and Game (20‐024), and USGS (24191).

We monitored the survival of one to two chicks chosen randomly from each brood (range = 7–23 chicks year^−1^) using 0.62‐g VHF radio transmitters, except for one brood in 2019 from which we captured two chicks >14 days after their estimated hatch date (see Appendix S1: Section S1.1). godwits are fully flight capable, or fledged, after ~28 days (Walker et al., 2020). However, because our radios had an expected lifespan of 21 days (range: 17–30 days; Holohil Systems, 2021), we considered chicks that survived to 21 days to have fledged. We confirmed mortalities when possible and assumed that radio tagged chicks had died after three consecutive days without relocating the chick. While some radios may have failed, we do not believe this occurred because the chicks known to have died did so, on average, by 3.6 days of age (SD: 8.3; range: 0–27) and we never resighted a chick that was presumed dead during weekly censuses of the bog and nearby areas (N. R. Senner, University of South Carolina, *unpublished data*).

### Resource monitoring

During 2009–2011, 2012, 2014–2016, 2017, and 2019, we monitored the biomass (i.e., resource abundance) and per‐capita body mass (i.e., resource quality) of invertebrates. In three periods, 2009–2011, 2014–2016, and 2019, we monitored invertebrates for an average of 67 days (range = 61–78 days) simultaneous with our godwit monitoring. Additionally, we monitored invertebrates, but not godwit nests, for 38 and 5 days in 2012 and 2017, respectively, as these were shortened seasons with limited crews.

Passive traps are a good proxy of resource availability for foraging shorebird chicks (Leung et al., 2018) and thus we collected invertebrates each day at 07:00 along two 50‐m transects consisting of five traps placed within mesic godwit breeding habitat (Brown et al., 2014; Senner et al., 2017). We used two trap styles: pitfall traps (10 × 15 cm) filled with 10 cm of 75% ethanol from 2009 to 2012, and modified malaise traps (see Leung et al., 2018) filled with 3 cm of 75% ethanol from 2014 to 2019. Invertebrate phenology measured from these transects does not differ from measurements taken across the study site and so we limited our analyses to these daily transects (Senner et al., 2017). We identified invertebrates to phylogenetic order and measured body lengths to the nearest 0.5 mm. We converted lengths to estimated dry mass using published, taxon‐specific length–mass relationships (Ganihar, 1997; Rogers et al., 1977). Invertebrate orders do not differ substantially in energy densities (James et al., 2012) and shorebird chicks generally select invertebrate prey based on their availability (Schekkerman & Boele, 2009). However, our data on invertebrate resources represent availability and not necessarily consumption by godwit chicks.

### Statistical analyses

Our statistical analyses had three goals: (1) quantify the variation in invertebrate resources over time; (2) determine the relationship between invertebrate abundance and quality, godwit chick growth, and godwit chick survival; and (3) identify the most appropriate model for relating variation in invertebrate abundance with population level measures of reproductive success. We describe the methods enabling us to meet these goals below.

### Interannual resource variation

To examine resource availability over the course of our study, we investigated how the (1) date of peak abundance; (2) inferred daily biomass (inferred dry mass; mg transect^−1^ day^−1^); and (3) daily median invertebrate body mass (per‐capita inferred dry mass; mg) changed across years. Because godwit chicks are gape‐limited and rarely consume larval invertebrates (Schekkerman et al., 2003), we restricted our analysis to only include invertebrates that are potential prey for godwits—that is adult invertebrates with lengths of 1.5–9 mm (Schekkerman & Boele, 2009). We excluded the shortened 2012 and 2017 seasons from our analyses of annual peak timing but included them in tests of daily biomass and invertebrate body mass.

We treated the transects as replicates, averaging the total daily biomass each day collected along each transect. To characterize the date of peak abundance, we estimated overall and order‐specific annual peaks using the first derivative of quadratic curves (day + day^2^) fit to the daily biomass within each year. Because shorebirds typically select prey according to availability, we built mixed‐effect models to estimate the linear trends of peak abundance (i.e., peak dates), daily invertebrate biomass, and daily invertebrate body mass for each order and overall. We included a random intercept for trap type in all our models using the lmer function (package *lme4*; Bates et al., 2020). In the daily biomass and daily invertebrate body mass models, we also included a random intercept of sample date to compare daily trends over the course of the study. We used the coefficient from each univariate mixed‐effect model to represent the temporal trend in the response variable. We performed all analyses in the R programming environment (v. 4.0.3, R Core Team 2020).

Shorebird young consume a wide diversity of prey items (Beintema et al., 1991). To identify potential changes in the composition of the invertebrate assemblage, we repeated the above analyses with each of the six orders that comprised 91.6% of all observed invertebrates: Araneae (20.5%), Hymenoptera (18.4%), Coleoptera (17.5%), Diptera (16.2%), Acari (11.3%), and Hemiptera (7.7%; Appendix S1: Figure S2). We excluded Collembola (8.3%), which are primarily aquatic, from our analyses as they are infrequent prey for godwit chicks and were also poorly recorded, due to their low frequency from 2009 to 2012. We standardized response variables by subtracting the mean and dividing by two SDs according to Gelman (2008), but report coefficients in their original units throughout the text. We considered response variables whose 95% confidence intervals did not include zero as biologically relevant.

### Chick growth and body condition

To predict the age‐specific mass of chicks, we modeled chick growth with a logistic growth function using the *nlme* package (Pinheiro et al., 2020). Although godwit chicks may be sexually dimorphic like closely allied species (Loonstra et al., 2018), we lacked data on each individual's sex and therefore pooled the sexes in our analyses. Non‐sex‐specific growth curves of Black‐tailed godwit chicks (*L. limosa*) have been found to overestimate the age and condition of female chicks (i.e., the larger sex), and female chicks tend to deviate negatively from the mean logistic growth coefficient (*K*) (Loonstra et al., 2018). We therefore accounted for the absence of sex‐specific information here by including (1) chick ID as a random intercept when modeling *K* (Appendix S1: Figure S3). We fixed the asymptotic growth coefficient (*A*_0_) to 249 g, the mean adult mass for both sexes (Senner et al., 2017). Next, we developed four separate growth models with either constant or annual (2) growth coefficients (*K*); and (3) inflection points (*T*_*i*_). We performed 100 iterations for each model and included site‐specific estimates from Senner et al. (2017) as starting values. We compared the candidate mixed‐effect models using conditional Akaike's Information Criterion (cAIC, package *cAIC4*) scores to account for uncertainty in the random effect structure (Säfken et al., 2021). We considered candidate models with ΔcAIC < 4 as candidates for the top model. We calculated a chick's body condition index (BCI) at each recapture by dividing their observed weight gain since last capture by the predicted weight gain over the same time from the top growth model.

To investigate how resource characteristics influenced chick growth, we modeled BCI in relation to resource abundance and quality in all years with godwit monitoring except 2014, which lacked sufficient mass‐at‐capture measurements. Because resource abundance and quality could have either an immediate or cumulative effect, we used fixed‐effect‐only generalized additive models to determine the timescale over which they influenced BCI: day of recapture or 1‐, 3‐, or 7‐day averages, and used the timescale with the lowest AIC_c_ (AIC corrected for small sample sizes; Burnham & Anderson, 2002) score in further analyses. We also determined whether random intercepts, study year, brood ID, or individual, improved fit, and found near‐zero random variance components in models with a random intercept. We therefore did not include group random terms in further analyses. We built a global generalized additive mixed model with a Gaussian error term that included (1) daily invertebrate biomass, (2) daily invertebrate body mass, and (3) hatch date as fixed effects (package *gamlss*; Rigby & Stasinopoulos, 2005). Last, we included a penalized cubic spline for (4) chick age to account for irregular sampling by interpolating between observation periods (Wood, 2017). Before interpreting model fit, we ensured that all covariates included in the same model showed minimal collinearity by confirming pairwise Pearson's correlation coefficients (r) below 0.7 (Appendix S1: Table S2). We compared models by cAIC to account for the penalized spline. When no model had a model weight (*w*_*i*_) > 0.90, we performed model averaging for (ΔcAIC < 4) and report conditional averaged coefficients (Burnham & Anderson, 2002; Säfken et al., 2021).

### Effect of resources on survival: Constant or age varying?

To determine how resource quantity and quality affected chick survival, we built a Bayesian hierarchical survival model (see Appendix S1: Section S1.2). We quantified the effects of covariates we hypothesized influence godwit chick survival. We constructed a logit‐linear mixed model to estimate the additive effects of (1) daily invertebrate biomass, (2) daily invertebrate body mass, (3) hatch date, and (4) age along with random intercepts for (5) brood ID, (6) year, and (7) study plot. We included age, rather than a measure of metabolic rate, to account for both increasing energy demands (Williams et al., 2007) and other age‐related foraging behaviors (e.g., functional responses), as well as the fact that different predators prey on godwit chicks of different ages (Wilde, Swift, & Senner, 2022). We averaged our continuous parameters across 3‐day periods (i.e., our relocation interval) and standardized all variables by subtracting the mean and dividing by two SDs (Gelman, 2008). We chose diffuse priors for all predictors (Normal[0, τ]) and constrained random intercepts close to zero (mean = N(0, 1000), SD = Uniform(0, 25)). We again checked for collinearity between additive covariates with a pairwise Pearson's correlation coefficient (Appendix S1: Table S3). To test how the effects of daily invertebrate body mass or biomass varied with chick age, we built separate age‐interaction models and compared them using Watanabe's Akaike Information Criterion (WAIC) to estimate the out‐of‐sample expectation based on the log pointwise posterior predictive density (Watanabe, 2010). We included the interaction from the model with the lower WAIC score in all further models. Last, to identify the top model, we performed model selection using the indicator‐variable approach (Link & Barker, 2006; see Appendix S1: Section S1.2).

We constructed models of daily chick survival using the *runjags* and *rjags* packages (JAGS 4.1.0; Denwood, 2016; Plummer, 2003). Our models accessed three parallel chains to perform 5000 iterations. We removed 600 and 1000 iterations for adaptation and burn‐in, respectively, with a one‐third thinning factor. We assessed model performance based on the values of a Gelman‐Rubin statistic<1.1 and chain mixing (Gelman & Rubin, 1992). For all tests, we report the beta coefficients in logit‐form, 95% credible interval, and Bayesian *p* value (probability of slope ≠ 0).

### Population match and reproductive success

To quantify population level mismatches, we built annual resource and consumer demand curves. We estimated the annual resource curve by calculating the daily proportion of invertebrate biomass in a given year (Kwon et al., 2019; Leung et al., 2018). Additionally, we built competing demand curves from the (1) peak demand and (2) whole demand models (Figure 2) to quantify the effect of dynamic consumer demands on godwit reproductive success (see Appendix S1: Section S1.3). We also estimated the (3) curve height in each year (i.e., cumulative resource availability) from the area under the resource curve. Last, we calculated the (4) difference in peak dates (i.e., synchrony) between the resource and peak demand fitted curves in each year from the point at which each curve's derivative was zero. From these four metrics, we built four univariate linear models relating the different measures to fledging rates. We estimated survival to 21 days and the associated SD from the daily survival rate estimates from our global Bayesian model using the Delta method (Powell, 2007). We compared among the four, fixed‐effect models by calculating model weights from their AIC_c_ and their *R* ^2^ values.

## RESULTS

We located 142 godwit nests from 2009 to 2019, of which 128 survived to hatch. We individually marked 349 chicks (2009–2011, *n =* 195; 2014–2016, *n* = 106; 2019, *n* = 48) and attached radios to 128 chicks from 102 distinct broods. On average, radio‐tagged chicks survived to 9.4 days (SD = 8.4 days, range = 0–21). We relocated radio‐tagged chicks an average of 4.3 times (SD = 2.71, range = 1–19; *n* = 778) and recaptured them 1.5 times (SD = 0.83; *n* = 103). In most cases of chick death (*n* = 89), we located a carcass (37%) or found a detached radio (20%) in habitats clearly suggestive of predators (e.g., on a gull nesting island) within an average of 2 days (range = 0–4 days) of the first failed relocation attempt (Wilde, Swift, & Senner, 2022).

### Interannual changes in resources

We recorded the body lengths of 69,598 adult invertebrates across 14 orders, 41,298 of which were potential godwit prey (i.e., 1.5–9 mm). Sample days showed wide variation in the invertebrate biomass (x® = 132.9 mg, range = 0–948.4 mg) and invertebrate body mass (x® = 1.5 mg, range = 0.2–13.4 mg). We found no change in the timing of the predicted peak dates of all invertebrates (β = −1.68 ± 3.08 days, 95% CI = −3.34, 5.50 days; $R_{m}^{2}$ = −0.02; $R_{c}^{2}$ = −0.05) or among the individual orders over the course of the study Appendix (S1: Figure S4, left). However, both daily invertebrate biomass (β = −2.49 ± 0.50 mg, 95% CI = −3.49, −1.51; $R_{m}^{2}$ = −0.18; $R_{c}^{2}$ = −0.35; Appendix S1: Figure S4, center) and daily invertebrate body mass (β = −0.33 ± 0.03 mg, 95% CI = −0.028, −0.37; $R_{m}^{2}$ = −0.13; $R_{c}^{2}$ = −0.26; Appendix S1: Figure S4, right) decreased at a rate of −2% and −5% per year, respectively. At the order level, only Acari became more abundant over time (β = 0.20 ± 0.02 mg, 95% CI = 0.15, 0.25; $R_{m}^{2}$ = 0.14; $R_{c}^{2}$ = 0.38), while all other taxa became less abundant (Appendix S1: Figure S4). Additionally, Araneae (β = −0.67 ± 0.09 mg, 95% CI = −0.49, −0.85; $R_{m}^{2}$ = −0.19; $R_{c}^{2}$ = −0.28), Diptera (β = −0.24 ± 0.02 mg, 95% CI = −0.20, −0.29; $R_{m}^{2}$ = −0.03; $R_{c}^{2}$ = −0.15), and Hemiptera (β = −0.23 ± 0.06 mg, 95% CI = −0.10, −0.35; $R_{m}^{2}$ = −0.11; $R_{c}^{2}$ = −0.16) showed consistent decreases in body mass over the course of the study.

Additionally, we found opposing trends in daily biomass during the early and late portions of the godwit breeding season. Days during the nest incubation period (16 May–6 June) from 2014 to 2019 had 83% higher invertebrate biomass than those from 2009 to 2012, but days during the chick‐rearing period (6 June–4 July) had 41% lower biomass. Meanwhile, invertebrate body masses from 2014 to 2019 were 42%–72% smaller than those from 2009 to 2012.

### Chick growth and body condition

We modeled godwit chick growth from 103 mass‐at‐capture measurements taken following the initial measures collected at hatch. We estimated age‐specific mass from our top‐performing growth model alone (*w*_*i*_ > 0.9; Appendix S1: Table S4). Chick growth differed among years, and our top‐performing fixed‐asymptote growth function included a random intercept of individual for the logistic growth coefficient (*K*) and annual inflection points (*T*_*i*_; Appendix S1: Figure S5).

**FIGURE 3:**
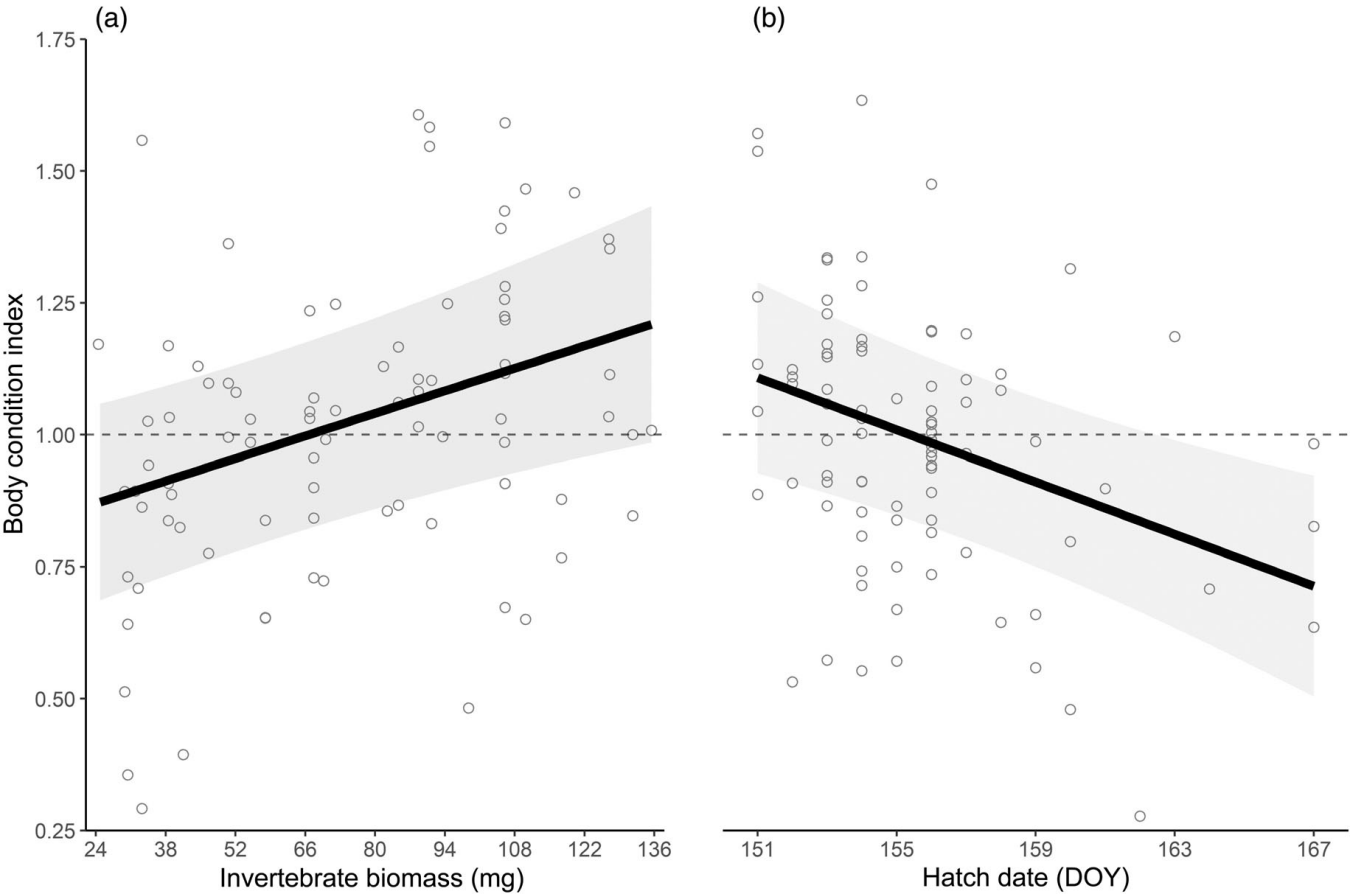
Effect of (a) daily invertebrate biomass (weekly average) and (b) hatch date (day of year with 1 = 1 January) on Hudsonian godwit chick body condition index (BCI). BCI >1 suggests above average growth and BCI <1 below average growth (BCI = 1, dashed). Predicted line (black) and 95% confidence interval (gray) are shown.

The fit of our global model was greatest using 7‐day averages of our continuous variables, daily biomass and daily median body mass, and no random intercept (Appendix S1: Table S5). Our top model predicting chick BCI (*n* = 98) included invertebrate biomass and hatch date with a smoothed age effect (*w*_*i*_ = 0.75; Appendix S1: Table S6). Chick growth improved with higher invertebrate biomass (β = 3.0 × 10^−3^ ± 1.1 × 10^−4^ mg^−1^, 95% CI = 8.7 × 10^−4^, 5.1 × 10^−3^; *R* ^2^_adj._ = 0.38; Figure 3a) but decreased with later hatch dates (β = −0.028 ± 0.010 days^−1^, CI = −0.009, −0.048; Figure 3b). Invertebrate body mass had no consistent effect on chick BCI despite a large mean effect size (Appendix S1: Figure S6). Exploratory analysis showed that body mass was only important for chicks older than 11 days. Chicks had 1%–17% higher body condition indices during periods with higher‐than‐average invertebrate biomass compared to periods with low invertebrate abundance. In terms of phenology, chicks grew better than expected if they hatched before 5 June but worse than expected thereafter.

### Effect of resources on survival: Constant or age varying?

Of the 128 godwit chicks in our study, we excluded six due to human‐caused mortality or instances when the radio fell off on the day of deployment. The mean DSR of the remaining 122 chicks was 86% ± 24%, meaning that 19.2% ± 33% survived to fledge, although this varied among years and broods (Appendix S1: Table S7).

The model with an age‐varying effect of invertebrate body mass (WAIC = 239.7, SE = 2.0) outperformed the model with an age‐varying effect of invertebrate biomass (WAIC = 562.5, SE = 19.5; Appendix S1: Table S8). We therefore used the former in our subsequent models. The constant effect of invertebrate biomass and the age‐varying invertebrate body mass effect had 79% and 85% posterior inclusion probabilities, respectively (Appendix S1: Table S9). We also included constant effects of age and invertebrate body mass to accompany the interaction term.

**FIGURE 4:**
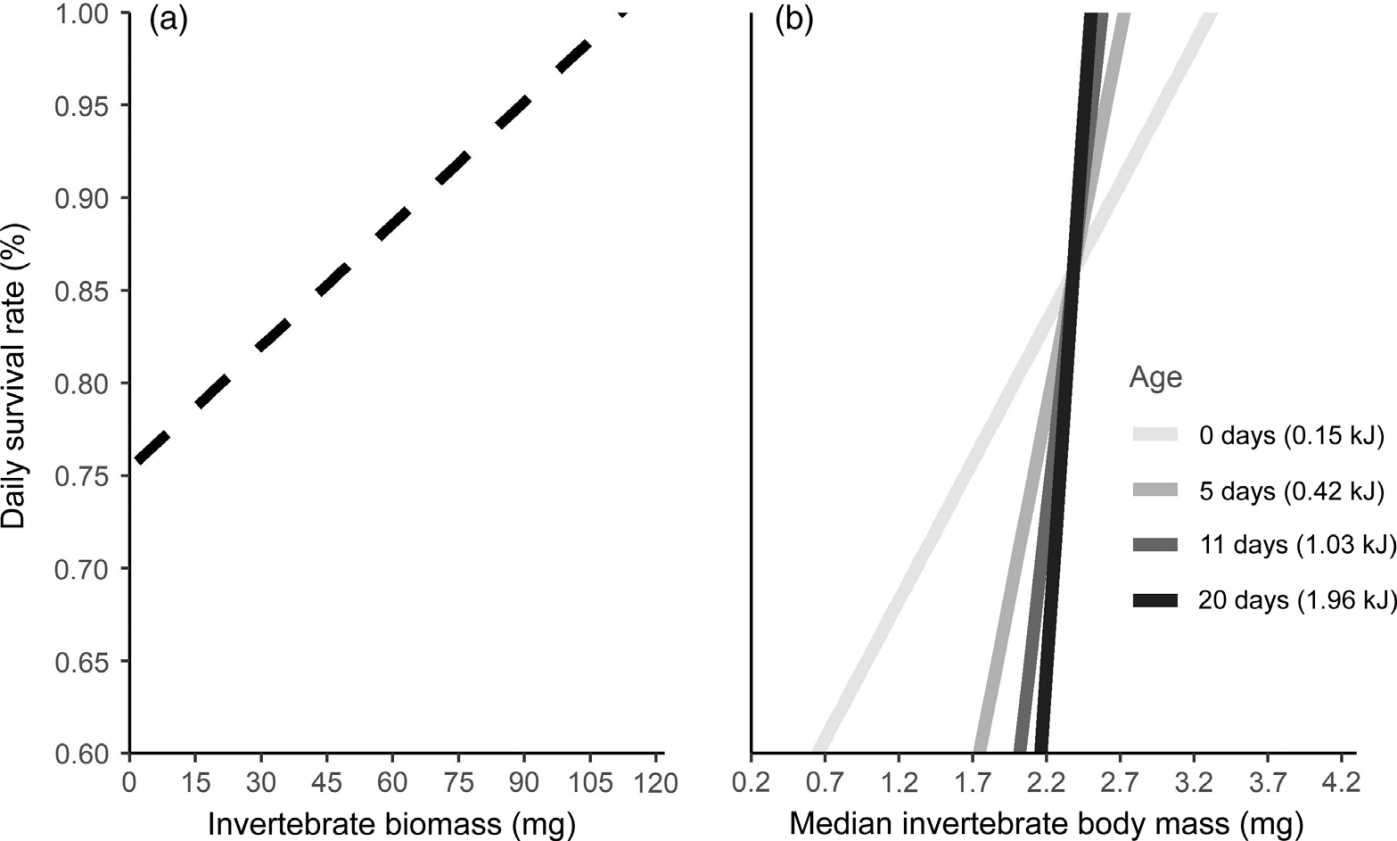
Effects of daily invertebrate biomass (a) and median body mass (b) on the survival of Hudsonian godwit chicks from the posterior mean estimates of a Bayesian hierarchical model. The effect of biomass (dashed) was constant, but that of size varied with age (shade of gray).

Chick survival improved with greater invertebrate biomass and larger invertebrate body mass, and the latter effect increased throughout development (Appendix S1: Table S10). Each 1% increase in daily invertebrate biomass (+1.5 mg) improved daily chick survival by 0.66% (Figure 4a), while each 1% increase in daily invertebrate body mass (+0.06 mg) led to a 1.02% increase in survival. The effect of invertebrate body mass further grew by 2.2% with each day a chick aged (Figure 4b). Age itself, however, had no consistent effect on survival.

### Population match and reproductive success

**FIGURE 5:**
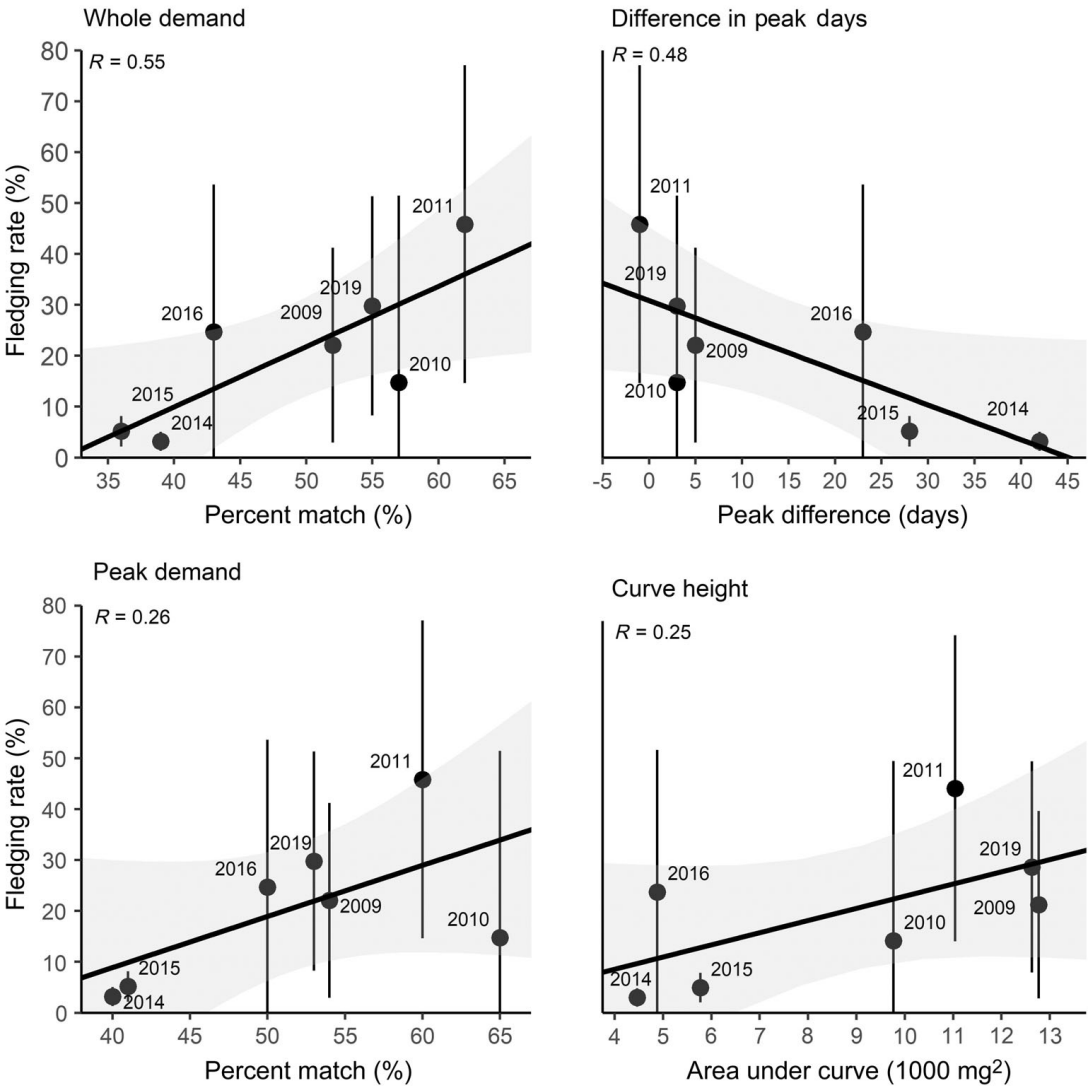
Correlation of annual fledging rates with measures of whole demand overlap (upper left), difference in peak dates (upper right), peak demand overlap (lower left), and curve height (lower right). Annual mean Hudsonian godwit fledging rates (black) and 95% confidence intervals (gray) were extrapolated from daily survival rates using the delta method. Univariate model correlation coefficients are displayed for each model.

The model fit for the whole demand curve (AIC_c_ = −300.1) was 25 times better than the peak demand curve (AIC_c_ = −248.7). godwits had, on average, 51.9% ± 9.2% overlap with resource phenology (in terms of annual proportions) according to the peak demand model, but 44.7% ± 11.6% overlap according to the whole demand model. Years also differed in curve height (x® = 8800 ± 3668 mg) and the difference in peak dates between the resource and demand curves (x® = 14.7 ± 16.36 days; Appendix S1: Figures S7, S8).

godwit fledging rates did not change linearly through time (Appendix S1: Table S11) but were lowest in 2014 and 2015. Those years had ~19% less overlap and ~28 day greater mismatches compared to the long‐term average. Mismatches on this scale resulted in 24% lower fledging rates and near complete reproductive failure for the population. Models differed in their ability to explain population level reproductive success but the whole demand model was best supported (Appendix S1: Table S12). The whole demand model explained 55% of the variation in godwit fledging rates (β = 1.19 ± 0.41; *R* ^2^_adj._ = 0.55; *w*_*i*_ = 0.43; Figure 5 upper left; Appendix S1: Figure S7). The “difference in peak dates” model performed similarly well (β = −0.68 ± 0.27; *R* ^2^_adj._ = 0.48; *w*_*i*_ = 0.36; Figure 5 upper right) but was 7% less likely to be the top model. Both the peak demand overlap (β = 1.00 ± 0.56; *R* ^2^_adj._ = 0.26; *w*_*i*_ = 0.11; Figure 5 lower left; Appendix S1: Figure S6) and curve height models (β = 2.49 ± 1.44; *R* ^2^_adj._ = 0.25; *w*_*i*_ = 0.10; Figure 5 lower right) were unlikely to be the top model given their low model weights and *R* ^2^ values.

## DISCUSSION

The disconnect between empirical results and the theoretical predictions of the match‐mismatch hypothesis have made it difficult to assess the effects of climate change‐induced phenological mismatches on consumer populations (Visser & Gienapp, 2019). To remedy this gap and help connect mismatches to demographic processes, we developed mismatch models that incorporate an age‐structured representation of consumer demand. Using this approach, we identified heretofore undetected individual and population level fitness effects of mismatches in the Alaskan breeding population of Hudsonian godwits (see Senner et al., 2017). Our study joins the growing literature suggesting that quantifying mismatches in terms of resource availability and consumer energetic demands can provide important nuance about the risks mismatches pose to consumer populations (Simmonds et al., 2020; Takimoto & Sato, 2020).

### More than asynchrony: Resource availability as the driver of mismatch effects

We found that resources affected godwit chick survival in two distinct ways: first, periods with reduced resource abundance resulted in poorer growth and lower survival and, second, access to larger invertebrates was increasingly important to the survival of older chicks. Our findings differ from those of a previous study in this system, which found no effects of limited resource availability on chick survival in the Alaskan godwit breeding population (Senner et al., 2017). While we had not previously investigated the influence of invertebrate body mass on godwit chicks, our contradictory conclusions likely stem from our use of hierarchical models that can approximate time‐varying effects on survival (Royle & Dorazio, 2009). Increasing energetic demands and changing foraging behaviors throughout development mean that the effects of resource limitation likely change over an individual's lifetime (Samplonius et al., 2016; Takimoto & Sato, 2020; Yang & Rudolf, 2010). Therefore, models that accommodate varying predictor effects may be key to clarifying how resource characteristics affect consumer fitness.

Having adequate resources during energetically demanding periods is a primary driver of animal fitness (Schekkerman & Visser, 2001). Given their high energetic demands and rapid development, chicks of shorebird species across the Arctic exhibit survival costs associated with reduced resource abundance (Saalfeld et al., 2019; Schekkerman et al., 2003). Accordingly, godwit chicks in our study grew better and had higher probabilities of survival during periods of higher‐than‐average invertebrate abundance. Although we detected effects of hatch date (i.e., phenology) on chick growth, these did not translate into an effect on survival. Our results therefore suggest that relating fitness measures to resource availability captures the effects of mismatches while requiring fewer assumptions about consumer–resource phenologies than synchrony‐based models (Durant et al., 2005).

In addition to the effects of resource abundance, the quality (i.e., median body mass) of invertebrates became increasingly important as godwit chicks required more energy. Optimal foraging theory predicts that consumers should select resources with the most energy content relative to foraging effort (Weterings et al., 2018). Black‐tailed godwit chicks, for instance, prioritize the rapid intake of small prey early in life, but switch to the slower intake of larger prey as they grow older (Schekkerman & Boele, 2009). While we did not observe foraging behaviors directly, we hypothesize that Hudsonian godwit chicks may make a similar transition and an increasing preference for larger prey could explain the higher costs of poor resource quality for older chicks. A preference for larger prey may allow older chicks to meet increasing energetic demands despite seasonal declines in resource abundance. Resource quality, while underrepresented in most mismatch studies, can thus have strong effects on consumer fitness, and including it in future studies can help elucidate the temporal dynamics of consumer–resource interactions.

Taken together, the additive effects of resource quantity and quality are likely to worsen in Beluga given the changes we observed in the invertebrate community. Climate‐induced reductions in resource availability are common across terrestrial and marine systems (Weterings et al., 2018). Arctic invertebrates, in particular, are simultaneously emerging earlier (Pearce‐Higgins et al., 2005), becoming less abundant, and decreasing in size (Jonsson et al., 2015) with increasing spring temperatures. Here, we found a linear decrease in the daily abundance and daily median body mass of invertebrates, as well as opposing trends in the abundance of invertebrates during the early and late portions of the godwit breeding season. Therefore, unless godwit chicks are able to compensate by prey‐switching to less abundant prey types (Samplonius et al., 2016), they may face increasingly untenable conditions as food becomes less abundant and poorer in quality.

More broadly, our results suggest that resource timing, quality, and quantity can act as concomitant drivers of phenological mismatches, and that their effects may be most apparent when placed in the context of the consumer life cycle (Samplonius et al., 2016). Thus, some individuals will encounter high‐quality resource conditions in years when they are “mismatched” (Kerby et al., 2012) or, conversely, low‐quality conditions when their demands are greatest. As a result, accounting for the variable effects of resource availability could improve our ability to document the true effects of mismatches that are otherwise difficult to detect.

### Modeling the demand–resource interaction clarifies the population effects of mismatches

Variation in godwit reproductive success at the population level was best explained by our whole demand model, although the simpler difference in peak dates model also performed well. Ramakers et al. (2020) argued that the assumptions of dates models‐ first, that individuals have the same energetic requirements and, second, that the measurements made on the resource capture the reality of the whole study area‐ make them more appropriate for empirical studies. We found, however, that by defining resource phenology in terms of seasonal proportions and directly modeling the differing energetic needs of consumers through time, overlap models can be made robust. Nevertheless, while estimates from overlap and dates models often correlate (Ramakers et al., 2020), either may outperform alternatives depending on a species' life history and degree of trophic specialization (Miller‐Rushing et al., 2010). Thus, while difference in peak dates models may suffice for godwits and other species with narrow, synchronous breeding phenologies or those that rely on singular resource pulses (Miller‐Rushing et al., 2010), they would likely perform poorly in species with highly variable nest initiation dates or those capable of multiple nesting events (Phillimore et al., 2016). Furthermore, difference in peak dates models could prove less accurate than overlap models when resource phenology is multimodal or lacks a clearly defined peak (Pearce‐Higgins et al., 2005; Samplonius et al., 2016). Because overlap models account for both the magnitude and duration of consumer‐resource interactions, they are more likely to capture mismatches as a disrupted interaction (Kerby et al., 2012). Overlap models are therefore likely more generalizable but using both overlap and difference in peak dates models could help when exploring how mismatches occur on a case‐by‐case basis.

Not all overlap models are equivalent, however, and overlap models have received mixed support (Ramakers et al., 2020). Whereas our peak demand model performed relatively poorly, our whole demand model explained the most variation in fledging rates among our suite of models. The difference between the two models' performance likely stems from the inability of the peak demand model to accurately capture consumer–resource interactions when individual level energetic demand is greatest, at the upper (i.e., right‐hand) tail of the consumer curve. Our results therefore show that incorporating additional nuance into the statistical concept of consumer phenologies can greatly improve overlap models (Lindén, 2018).

The need to accurately identify mismatches is made most clear by the accumulating evidence for variable and nonlinear responses by consumer populations to mismatches (Phillimore et al., 2016; Visser & Both, 2005). So called “tipping points,” thresholds past which an effect abruptly changes, buffer consumer populations from the negative impacts of moderate mismatches and may contribute to the lack of consistent responses to mismatches across consumer populations (Simmonds et al., 2020). In this population of godwits, we found that greater population level mismatches consistently drove poorer fledging success, but that there may be thresholds past which the effects are most severe. For instance, the degree of mismatch in 2014 and 2015 resulted in near complete reproductive failure for the population. Similarly low fledging rates for Hudson Bay breeding godwits, which are mismatched by 11 days on average (Senner et al., 2017), suggest that, for godwits, this tipping point may exist when populations are mismatched by more than ~10 days or have less than 40% overlap with the resource curve.

Importantly though, the 2014 and 2015 seasons in Beluga coincided with a period of anomalous and prolonged near‐surface warming in the northeastern Pacific called the “blob” (Auth et al., 2018). Thus, while the conditions in these atypical years may provide useful insights into potential outcomes of a warming climate on coastal communities in the region, mismatches of this magnitude may not become the norm. Beluga godwits have been able to advance their timing of migration and reproduction in response to recent long‐term, linear warming trends (Senner, 2012; Senner et al., 2017). Nonetheless, their ability to do so into the future will depend on whether the cues godwits use to time their annual cycle remain predictive of resource phenology on the breeding grounds. The significant spring warming and earlier snow disappearance dates projected for the sub‐Arctic, for instance, mean that godwits and other migratory populations may soon face accelerating, potentially nonlinear warming to which they have limited capacity to respond.

### Conclusions

By modeling mismatches directly in terms of dynamically interacting resource and consumer needs, we stand to adopt a more powerful definition of mismatches and be better able to identify the circumstances under which consumer populations perform poorly. Our work also illustrates the role of a consumer's stage in development in shaping their changing response to resource availability over time and helps explain the empirical‐theoretical disconnect in phenological studies. Importantly, our models are transferrable to other systems, whereby remotely sensed indices and knowledge of a population's age structure could approximate resource availability and energetic requirements, respectively, when more detailed data are otherwise unavailable. Finally, we show how treating mismatches as an outcome of both consumer demands and resource dynamics provides insight into the structure of individual level effects and the mechanism behind population level responses. Replacing the synchrony‐based definition of mismatches with one explicitly recognizing the interaction between consumer needs and resource availability may be critical to monitoring and conserving animal populations in an uncertain future.

## AUTHOR CONTRIBUTIONS

Luke R. Wilde and Nathan R. Senner conceived of the study. All authors collected field data. Luke R. Wilde analyzed the data and wrote the manuscript. All authors contributed to revisions and have given final approval for publication.

## CONFLICT OF INTEREST

The authors declare no conflict of interest.

## Supporting information


Appendix S1

## Data Availability

Data (Wilde et al., 2022b) are available in Dryad at https://doi.org/10.5061/dryad.x69p8czh0. Code (Wilde et al., 2022a) is available on Zenodo at https://doi.org/10.5281/zenodo.6149631.
