# Supplementary material for: Dynamic sensitivity to resource availability influences population responses to mismatches in a shorebird
Source: Ecology. 2022 Jun 12;103(9):e3743. doi: 10.1002/ecy.3743 (PMC9539520; doi:10.1002/ecy.3743)
Supplement: Supplementary file 1 — Appendix S1 [file ECY-103-e3743-s001.pdf]

Appendix S1

Wilde, Luke R., Josiah E. Simmons, Rose J. Swift, and Nathan R. Senner. 2022. Dynamic sensitivity to resource availability influences population responses to mismatches in a shorebird. Ecology.

Section S1.1: *Study area and godwit chick monitoring*

We attached a 0.62 g radio transmitter (Holohil Systems Ltd.) above the uropygial gland by clipping the feathers and using skin-safe, cyanoacrylate glue to attach the radios directly to the skin. We relocated each radio tagged chick every 2–3 days by walking the entirety of the study plot and recording telemetry azimuths from <100 m of the tending parent(s)'s location. When no parents were present, we scanned for signals in all directions from a chick's last known location. We attempted to recapture radio tagged chicks weekly to reapply glue and measure their body mass to the nearest gram, minus the transmitter weight. We elected for weekly captures based on Sharpe et al. (2009), who found weekly sampling to be adequate for accurate assessment of growth rates while minimizing the potential for adverse handling effects.

Section S1.2: *Data structure and Bayesian model selection*

We constructed daily encounter histories for all individuals, beginning with an individual's hatch date and ending with their expected fledging date. Because we assumed the chicks that we could not relocate for three consecutive days were dead, we included two days of unknown fate to allow for Markov chain Monte Carlo (MCMC) prediction. We modelled encounter histories as a Bernoulli variable and assumed fates were known.

We assigned a Bernoulli variable (weights) with a 0.5 prior to each predictor to model its inclusion (1) or absence (0) from each MCMC sample. We maintained an equal number of parameters across samples by fixing the model variance, $\tau = K$ * Gamma(3.29, 7.8), for all parameters, where K is the number of parameters (Link & Barker, 2006). The posterior mean of the weight indicator is evidence for inclusion in the model. We calculated Bayes factors (BF) from predictor weights (Link & Barker, 2006) and included predictors with BF>3 in our top

model along with their random intercepts. If an interaction term was selected for the top model, we included both additive terms included in the interaction in the top model.

Section S1.3: *Constructing peak and whole demand curves*

Following Kwon et al. (2019), we estimated the (1) *peak demand* of godwit chicks by calculating the number of all hatched godwit chicks expected to be 11-days old (i.e., age of peak growth rate; Senner et al. 2017) for each day of the year. We then converted both the daily values of invertebrate biomass (hereafter, 'resource curve') and counts of 11-day old chicks to their annual proportions. As a test of how a more nuanced definition of mismatches can improve detection of their effects, we developed a (2) *whole demand* model. We multiplied the maximum number of chicks of each age per day of the year by mass-specific estimates of resting metabolic rate (RMR; Kilojoules per day, kJ d^{-1}) in godwit chicks (Williams et al., 2007). We estimated each chick's RMR from published mass-specific estimates of godwit chicks (Williams et al., 2007), and age-specific mass estimates from our top-performing growth curve. RMR approximates the amount of energy individuals use to maintain homeostasis and therefore represents an individual's minimum energetic requirement independent of other factors (i.e., thermal environment). We then estimated the cumulative, minimum energetic requirements of all chicks per day of the year and converted these to annual proportions to produce the whole demand curve.

We modelled the shape of the peak demand, whole demand, and resource curves using separate generalized additive models with a quadratic time function – day+day^2 (Kwon et al. 2019). We restricted the analyses to 10 May–10 Jul for comparison among study periods, which otherwise differed in length (Appendix S1: Table S3). We approximated error terms as a

gaussian distribution (~N[μ,σ]) and zero-inflated beta distributions (~ zBeta[z|α, β]) for the peak demand and whole demand curves, respectively, and a beta distribution (~Beta[α, β]) for the resource curve, all with logit-link functions. We fit the resource curve with a penalized spline (k=10) to estimate mean predicted values for each day of the year while capturing the modality of the resource curve (Vatka et al., 2016). We then estimated the degree of overlap between the peak demand or whole demand curves and the resource curve by calculating the proportional area overlap using the *integrate.xy* function ('sfsmisc'; Maechler 2020).

Table S1. Summary of season lengths and the observed Hudsonian godwit hatching dates and model predicted invertebrate resource peak from 2009 to 2019 near Beluga River, AK. The resource peak was calculated as the day with the smallest first derivative along the day + day^2 curve. Neither godwits or invertebrates were monitored for the full 2012 or 2017 season.

Year	*Chicks hatched*	*Season length*	*Mean hatch date (± SD)*	*Predicted resource peak*
2009	69	3 May–10 Jul	5 Jun (± 5.5 d)	5 Jun
2010	60	3 May–10 Jul	11 Jun (± 9.4 d)	8 Jun
2011	87	3 May–10 Jul	7 Jun (± 5.0 d)	14 Jun
2012	32	8 May–5 Jun	9 Jun (± 5.0 d)	--
2014	31	9 May–13 Jul	9 Jun (± 5.6 d)	10 May
2015	62	3 May–10 Jul	6 Jun (± 3.2 d)	31 May
2016	38	1 May–10 Jul	5 Jun (± 4.0 d)	20 May
2017	--	11 May–19 May	--	--
2019	56	6 May–26 Jul	7 Jun (± 5.3 d)	11 Jun

Table S2. Estimates of Pearson's correlation coefficient (r) among pairs of fixed effect covariates in a generalized additive mixed model predicting godwit chick body condition index estimates collected near Beluga River, AK from 2009 to 2019.

Variable 1	*Variable 2*	*r*	*lower*	*upper*
Size	Insect	0.22	0.01	0.41
Size	Hatch	0.27	0.07	0.46
Insect	Hatch	0.04	-0.17	0.24

Size: daily median invertebrate body mass; Biomass: daily invertebrate biomass

Table S3. Estimates of Pearson's correlation coefficient (r) among pairs of fixed effect covariates in a Bayesian hierarchical model on the survival of Hudsonian godwit chicks living near Beluga River, AK (2009 to 2019). Coefficients were measured to check collinearity between predictors in a global model prior to model selection procedures.

Variable 1	*Variable 2*	*r*	*lower*	*upper*
Size	Age	-0.006	-0.04	0.03
Size	Insect	0.32	0.78	0.36
Size	Hatch	-0.05	-0.09	-0.01
Insect	Age	-0.03	-0.08	0.02
Insect	Hatch	0.07	0.02	0.11
Hatch	Age	-0.04	-0.06	-0.01

Age: number of days since hatch; Hatch: individual chick's hatch date; Size: daily median invertebrate body mass; Biomass: daily invertebrate biomass

Table S4. Model selection table of logistic models predicting godwit chick mass from weekly captures (*n*=481) of godwit chicks near Beluga River, AK from 2009 to 2019. Initial values were set prior to modeling: inflection point inflection point (T_i)=10.7, logistic coefficient (K)=0.12, Asymptotic mass=249 (*sensu*, Senner et al. 2017). The logistic growth coefficient (*K*) was allowed to vary by a random intercept for chick identity (ID). Parameter estimates were averaged from 100 iterations. cAIC= conditional Akaike's Information Criterion.

Model no.	*Variable*	*Value*	*ΔcAIC*	*log-likelihood*	*Model weight (w_i)*	*No. parameters*
2	T_i<br>K	~Year<br>~1	0	-542.801	0.96	9
1	T_i<br>K	~Year<br>~Year	18.950	-540.068	0.04	14
3	T_i<br>K	~1<br>~Year	68.771	-570.411	>0.01	9
4	T_i<br>K	~1<br>~1	83.238	-564.476	>0.01	4

Table S5. Model selection table of the timescale of continuous covariate in our global generalized additive model (no random effects) to predict the body condition index (BCI) of godwit chicks near Beluga River, Alaska from 2009 to 2019. Timescale is the period over which the continuous fixed effect variables – daily invertebrate biomass and daily median invertebrate body mass – in the global model were averaged for model smoothing. AIC_c = Akaike's Information Criterion corrected for small sample sizes.

Timescale				
	ΔAIC_c	*Deviance*	*Model weight (w_i)*	*log-Likelihood*
7-day avg.	0	12.7	0.69	-39.6
3-day avg.	2.3	13.0	0.22	-40.8
day of	4.7	134	0.06	-42.0
1-day avg.	6.4	13.6	0.03	-42.8

Table S6. Comparison by conditional Akaike's Information Criterion (cAIC) value among candidate models with predictor variables of Hudsonian godwit chick growth (*n*=89) from 2009 to 2019 near Beluga River, AK. Samples excluded chicks from 2014, which lacked recaptures. Growth was estimated from body condition index (BCI) scores obtained from weekly captures. Continuous predictors were averaged over a 7-day period prior to BCI estimation. Inclusion in a model is indicated by a beta coefficient for predictors and plus signs (+) for smoothing terms. Intercept only model is indicated in grey.

Intercept	*Invertebrate biomass*	*Hatch date*	*Invertebrate body mass*	*s(Chick age)*	*df*	*log-likelihood*	*cAIC*	*ΔcAIC*	*model weight*
4.923	0.003	-0.027		+	12	-10.938	45.77098	0	0.653382
5.471	0.003	-0.031	0.042	+	13	-10.498	46.14279	0.37181	0.260169
4.445		-0.023		+	11	-15.401	53.37417	7.603192	0.032552
0.74	0.003			+	11	-15.748	54.06789	8.296912	0.024161
5.101		-0.028	0.05	+	12	-14.841	53.57628	7.8053	0.015275
0.788	0.003		-0.018	+	12	-15.624	55.1434	9.37242	0.0072
0.926				+	10	-18.592	58.36293	12.59195	0.005626
0.942			-0.005	+	11	-18.590	59.75195	13.98097	>0.001
5.641	0.003	-0.033	0.081		4	-30.051	71.62525	25.85427	>0.001
4.657	0.003	-0.025			4	-31.603	74.73013	28.95915	>0.001
5.988		-0.034	0.091		3	-33.479	76.67619	30.90521	>0.001
0.691	0.003				3	-34.689	79.09615	33.32517	>0.001
4.902		-0.026			3	-35.282	80.28225	34.51127	>0.001
0.622	0.003		0.025		3	-34.534	78.78657	33.01559	>0.001
0.926					1	-38.144	82.24242	36.47145	>0.001
0.83			0.033		2	-37.887	83.63367	37.86269	>0.001

Intercept	*Invertebrate biomass*	*Hatch date*	*Invertebrate body mass*	*s(Chick age)*	*df*	*log-likelihood*	*cAIC*	*ΔcAIC*	*model weight*
4.923	0.003	-0.027		+	12	-10.938	45.77098	0	0.653382
5.471	0.003	-0.031	0.042	+	13	-10.498	46.14279	0.37181	0.260169
4.445		-0.023		+	11	-15.401	53.37417	7.603192	0.032552
0.74	0.003			+	11	-15.748	54.06789	8.296912	0.024161
5.101		-0.028	0.05	+	12	-14.841	53.57628	7.8053	0.015275
0.788	0.003		-0.018	+	12	-15.624	55.1434	9.37242	0.0072
0.926				+	10	-18.592	58.36293	12.59195	0.005626
0.942			-0.005	+	11	-18.590	59.75195	13.98097	>0.001
5.641	0.003	-0.033	0.081		4	-30.051	71.62525	25.85427	>0.001
4.657	0.003	-0.025			4	-31.603	74.73013	28.95915	>0.001
5.988		-0.034	0.091		3	-33.479	76.67619	30.90521	>0.001
0.691	0.003				3	-34.689	79.09615	33.32517	>0.001
4.902		-0.026			3	-35.282	80.28225	34.51127	>0.001
0.622	0.003		0.025		3	-34.534	78.78657	33.01559	>0.001
0.926					1	-38.144	82.24242	36.47145	>0.001
0.83			0.033		2	-37.887	83.63367	37.86269	>0.001

Table S7. Group levels (i.e., random intercepts) from a Bayesian hierarchical model predicting the daily survival rate of Hudsonian godwit chicks from 2009 to 2019 near Beluga River, AK. $\hat{R}$ is the Gelman–Rubin statistic where $\hat{R}<1.1$ is evidence of convergence (Gelman & Rubin, 1992). Individual histories were grouped by study year, brood ID, and study plot.

Group level	*Posterior Mean (SD)*	*95% Credible Interval*	*Effective Sample Size*	$\hat{R}$
2009	0.05 (13.64)	-28.12, 30.87	14279	1.0
2010	0.01 (13.67)	-28.99, 29.99	15274	1.0
2011	0.1 (13.49)	-30.53, 27.12	15000	1.0
2014	-0.15 (13.37)	-28.87, 27.64	15000	1.0
2015	0.05 (13.6)	-28.91, 28.86	15167	1.0
2016	0.08 (7.13)	-23.60, 2.56	14041	1.1
2019	0.11 (6.10)	-12.08, 11.35	14901	1.2
2009GN001	0.14 (8.86)	-17.30, 18.24	15000	1.0
2009GN002	0.06 (8.76)	-17.72, 17.84	16088	1.0
2009GN003	-0.08 (8.67)	-17.92, 16.96	14798	1.0
2009GN007	0.11 (8.69)	-17.57, 17.56	16964	1.0
2009GN010	-0.02 (8.73)	-17.27, 17.91	15639	1.0
2009GN012	0.11 (8.56)	-16.36, 18.09	14182	1.0
2009GN014	0.02 (8.76)	-18.02, 17.25	14961	1.0
2009GN018.2	0 (8.73)	-17.93, 17.00	14634	1.0
2009GN022	-0.01 (8.65)	-18.94, 16.39	15000	1.0
2009GN027	-0.01 (8.67)	-17.87, 16.76	14758	1.0
2009GN0282	0.08 (8.63)	-17.23, 17.50	15000	1.0
2009GN044	-0.1 (8.71)	-17.64, 17.27	14861	1.0
2009GN045	-0.1 (8.78)	-18.39, 16.86	16001	1.0
2009GN046	-0.2 (8.70)	-18.62, 16.41	14546	1.0
2009GN047	0.0 (8.59)	-16.63, 17.95	15000	1.0
2009GN049	0.03 (8.62)	-17.17, 17.34	15000	1.0
2010GN11	-0.02 (8.70)	-17.03, 17.74	14803	1.0
2010GN47	0.02 (8.81)	-18.13, 17.77	14507	1.0
2010GN58	-0.06 (8.77)	-18.62, 17.25	14934	1.0
2010GN61	-0.04 (8.75)	-18.04, 17.51	14517	1.0
2010GN62	0.02 (8.70)	-17.51, 17.35	14532	1.0
2010GN63	-0.06 (8.70)	-17.69, 17.42	15466	1.0
2010GNGPM	-0.1 (8.59)	-18.01, 16.85	15080	1.0
2010GNHUYU	-0.01 (8.67)	-18.03, 17.33	15000	1.0
2010GNPE	-0.01 (8.74)	-17.29, 18.19	14097	1.0
2010GNUL	0.01 (8.78)	-18.50, 16.97	15142	1.0
2010GNXEXY	0.1 (8.63)	-16.61, 17.69	15418	1.0
2010GNYN2	0.07 (8.64)	-17.32, 17.49	16213	1.0
2010GNYTXL	-0.01 (8.51)	-18.03, 16.35	14345	1.0

2011GN13	-0.01 (8.67)	-17.26, 17.53	15000	1.0
2011GNAPAU	-0.07 (8.74)	-18.48, 16.33	15215	1.0
2011GNC4T6	-0.09 (8.70)	-17.62, 17.25	15352	1.0
2011GNC8J2	0 (8.71)	-17.68, 17.39	15627	1.0
2011GNCT	-0.03 (8.65)	-17.23, 17.63	14886	1.0
2011GNE5E9	-0.01 (8.66)	-18.19, 16.62	16547	1.0
2011GNEAE7	0.02 (8.71)	-17.49, 17.98	15237	1.0
2011GNEE	-0.1 (8.68)	-17.61, 17.29	15149	1.0
2011GNH7T2	-0.02 (8.79)	-18.41, 17.55	15870	1.0
2011GNH8LO	-0.02 (8.69)	-17.94, 17.17	15000	1.0
2011GNJ5K7	-0.06 (8.63)	-17.96, 16.89	15812	1.0
2011GNJ6J0	-0.01 (8.76)	-17.60, 17.41	15054	1.0
2011GNJTMU	-0.15 (8.72)	-17.57, 17.3	14835	1.0
2011GNK0PM	-0.02 (8.69)	-18.08, 16.74	15140	1.0
2011GNK4T7	0.04 (8.68)	-17.54, 17.80	14034	1.0
2011GNM2P2	0.06 (8.59)	-17.17, 17.36	15133	1.0
2011GNM3U0	-0.05 (8.56)	-17.72, 16.90	14778	1.0
2011GNN8X3	0.05 (8.85)	-17.29, 18.38	14942	1.0
2011GNTANA	-0.1 (8.67)	-17.42, 17.53	15048	1.0
2011GNV9C0	0.1 (8.66)	-17.55, 17.17	15378	1.0
2011GNX50H	-0.04 (8.69)	-17.13, 17.56	17680	1.0
2011GNX6H3	0.0 (8.75)	-17.21, 18.30	15552	1.0
2011GNY9L6	0.05 (8.69)	-17.11, 17.55	14683	1.0
2014BHD17	-0.09 (8.65)	-16.81, 18.31	15443	1.0
2014BHD19	0.06 (8.56)	-17.82, 16.69	15000	1.0
2014BJL17	0.03 (8.74)	-17.33, 17.81	14793	1.0
2014BJL18	0.08 (8.62)	-16.91, 17.5	15561	1.0
2014BJL19	0.01 (8.74)	-16.72, 18.86	14404	1.0
2014BJL25	0.12 (8.63)	-18.14, 16.59	15273	1.0
2014GJM06	-0.11 (8.65)	-16.67, 18.44	15000	1.0
2015GJM05	0.08 (8.60)	-16.51, 18.20	15402	1.0
2015GJM18	-0.03 (8.65)	-17.03, 18.23	15705	1.0
2015GJM35	0.09 (8.66)	-17.71, 17.12	15000	1.0
2015GJM36	-0.02 (8.59)	-17.06, 17.39	14401	1.01
2015JAK05	0.04 (8.66)	-17.56, 17.46	14887	1.0
2015JAK21	0.01 (8.71)	-16.73, 18.05	16195	1.0
2015JMH10	0.01 (8.76)	-18.48, 16.96	15801	1.0
2015JMH20	0.1 (8.82)	-17.38, 18.13	15056	1.0
2015JMH28	0.01 (8.81)	-18.11, 17.84	16209	1.0
2015KJP18	-0.01 (8.68)	-18.69, 16.75	14590	1.0
2015KJP44	0.08 (8.63)	-16.9, 17.92	15242	1.0
2015RJS05	-0.03 (8.57)	-18.11, 16.63	14870	1.0
2015RJS05	0.01 (8.62)	-16.81, 17.70	15000	1.0
2015U1MUV	-0.08 (8.74)	-18.08, 17.02	15000	1.0
2016KRS48	-9.52 (7.63)	-23.78, 7.31	12247	1.02
2016LKF04	0.56 (8.09)	-15.47, 16.61	13272	1.0
2016LKF22	0.01 (7.83)	-14.38, 15.86	15014	1.01
2016MLS14	0.93 (7.97)	-15.23, 16.6	13951	1.0
2016MLS37	1.58 (6.71)	-11.8, 15.43	12726	1.01

2016RIG15	-0.33 (6.32)	-12.24, 13.79	12952	1.01
2016RJS04	0.40 (7.66)	-13.98, 16.00	12602	1.0
2016RJS07	0.16 (7.88)	-14.45, 16.73	12428	1.01
2016RJS10	-3.46 (5.03)	-14.37, 6.13	12259	1.08
2016RJS16	3.0 (7.09)	-11.12, 17.81	12935	1.02
2019GB01	-7.09 (3.08)	-13.5, -1.65	14744	1.01
2019GB02	-2.31 (4.56)	-11.09, 7.26	14948	1.05
2019GB03	-7.96 (4.41)	-15.49, 1.97	13044	1.07
2019GN01	1.60 (4.38)	-6.11, 11.44	15159	1.08
2019GN02	10.11 (5.97)	-0.66, 22.54	15057	1.02
2019GN03	-8.88 (4.40)	-16.79, 0.63	13173	1.07
2019GN05	2.89 (6.6)	-7.96, 16.62	14273	1.04
2019GN06	8.15 (5.67)	-2.12, 19.74	14424	1.01
2019GN08	0.65 (2.99)	-5.79, 5.92	13055	1.01
2019GN09	6.69 (6.41)	-6.64, 19.79	12900	1.01
2019GN10	-3.69 (6.79)	-15.05, 11.02	12128	1.01
2019GN11	-5.76 (3.11)	-12.24, 0.12	13962	1.02
2019GN12	8.23 (5.62)	-1.68, 19.93	14157	1.01
South Plot	0.26 (4.43)	-8.25, 9.21	14071	1.16
North Plot	3.28 (4.78)	-3.97, 14.22	12493	1.02

Table S8. Model selection of competing hierarchical models with age-varying effects (i.e., interaction effect with age) of invertebrate body mass or invertebrate biomass from logit-link, binomial regressions performed in JAGS on the daily survival of godwit chicks near Beluga River, AK from 2009 to 2019. Watanabe's-Akaike Information Criterion (WAIC) posterior probabilities were estimated from a log-likelihood matrix (dimensions: [73200,39]), where the model with a lower WAIC posterior mean and variance is best supported.

Age-varying interaction: main effect term	*Statistic*	*Posterior mean*	*Posterior standard error*
Invertebrate body mass	ELPD	-119.8	1.0
	PEff	5.4	0.9
	WAIC	239.7	2.0
Invertebrate biomass	ELPD	-281.3	3.7
	PEff	6.8	2.7
	WAIC	562.5	19.5

(ELPD=expected log pointwise predictive density, PEff=effective number of parameters, WAIC=Watanabe's-Akaike Information Criterion)

Table S9. Bayesian model selection on variables in a global logistic model predicting daily survival rate in godwit chicks near Beluga River, AK from 2009 to 2019. Predictors were selected using the indicator-variable approach, in which posterior inclusion probabilities (weights) and Bayes Factors (BF) were estimated from a Bernoulli variable associated with each predictor. Variables of the global model with BF > 3 and their component parts (i.e., interaction terms) were included in the top model.

Variable	Weight, global	BF, global	Weight, top	BF, top
Age	0.53	1.125	0.52	1.081
Size	0.54	1.177	0.52	1.068
Hatch	0.54	1.175	-	-
Biomass	0.79	3.730	0.77	3.340
Age × Size	0.85	5.614	0.80	4.042

Age: number of days since hatch; Hatch Date: individual chick's hatch date; Size: median daily invertebrate body mass; Biomass: daily invertebrate biomass

Table S10. Standardized effect of variables on the survival rates of godwit chicks near Beluga River, AK from 2009 to 2019. Posterior probabilities were estimated from a hierarchical model (n=122, posterior samples=5000) with both survival and stochastic model components.

Predictor	Mean (SD)	95% Credible Interval	Pr ≠ 0
Intercept	-2.388 (5.253)	-13.10, 7.17	0.35
Age	-0.001 (0.201)	-0.41, 0.38	0.48
Size	0.331 (0.185)	-0.03, 0.71	0.96
Biomass	0.26 (0.169)	-0.08, 0.58	0.87
Age × Size	0.679 (0.103)	0.48, 0.88	1.00

Age: number of days since hatch; Size: median daily invertebrate body size; Biomass: daily invertebrate biomass

Table S11. Seasonal daily survival rates (DSR) of Hudsonian godwit chicks (*n*=122) in Beluga River, AK among study years. DSR estimates from a Bayesian hierarchical model were extrapolated to 21 d as an estimate of percent fledged, and associated delta error is reported.

Year	*No. chicks*	*DSR (mean)*	*Standard deviation*	*95% credible interval*	*Estimated % fledged (± Delta error)*
2009	16	0.931	0.088	0.871, 0.964	22.11 (±19.13)
2010	16	0.913	0.178	0.810, 0.963	14.75 (±36.69)
2011	24	0.964	0.072	0.929, 0.982	45.84 (±21.22)
2014	7	0.849	0.174	0.672, 0.939	3.18 (±1.88)
2015	17	0.868	0.139	0.781, 0.924	5.16 (±3.00)
2016	20	0.936	0.097	0.890, 0.963	24.71 (±28.95)
2019	22	0.944	0.070	0.906, 0.967	29.79 (±21.54)

Table S12. Model selection table comparing univariate linear models of population mismatch models predicting seasonal fledging rates in a population of Hudsonian godwits near Beluga River, AK from 2009 to 2019. AIC_c=Akaike's Information Criterion corrected for small sample sizes. R^2 is the correlation coefficient.

Model	***logLikelihood***	AIC_c	ΔAIC_c	***Model weight (*** w_i ***)***	R^2
Whole Demand	-25.14	64.27	0	0.43	0.55
Difference in peak dates	-25.31	64.63	0.35	0.36	0.48
Peak Demand	-28.54	67.08	2.81	0.11	0.26
Curve height	-26.62	67.23	2.96	0.10	0.25

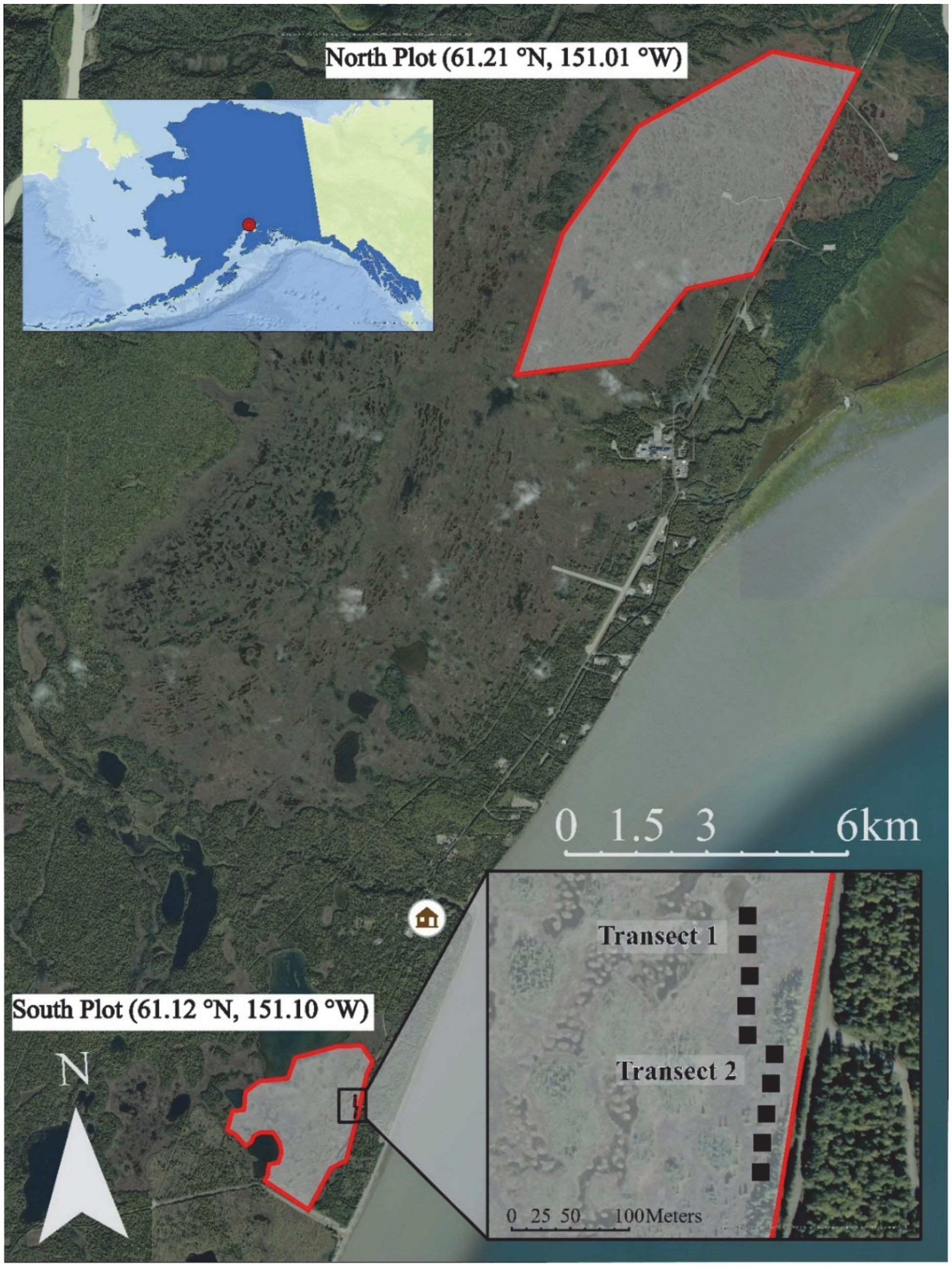

Figure S1. Map of North and South plots (red polygons) near the township of Beluga River, AK (house icon). (Inset top left) The study areas (red point) is located in southcentral AK on the west coast of the upper Cook Inlet. (Inset bottom right) The two, 50-m transects for daily invertebrate capture using pitfall traps (2009–2011) or modified malaise traps (2014–2016, 2019). Transects

were placed according to Arctic Shorebird Demographic Network protocols (Brown et al. 2014). Basemap images are the intellectual property of Esri and are used herein under license.
Copyright © 2014 Esri and its Licensors. All rights reserved. Additional data sources: U.S. Census Bureau 2018.

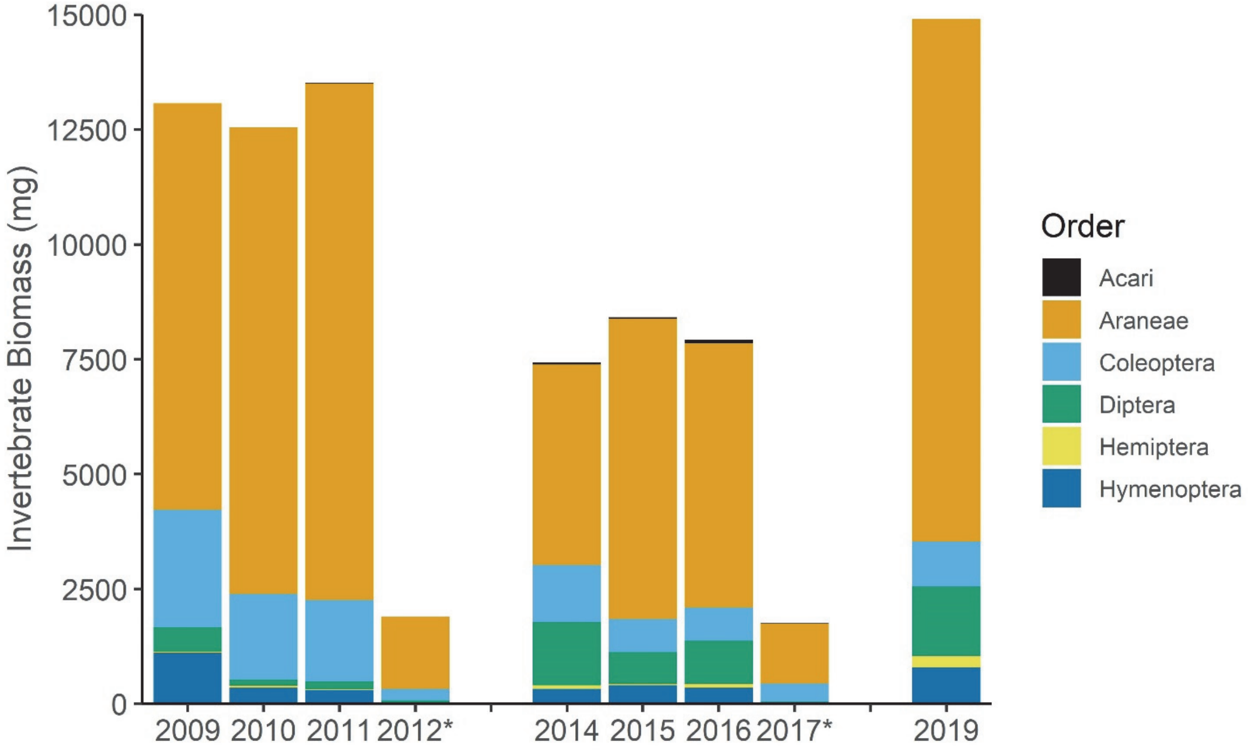

Figure S2. Interannual comparison of the available biomass and composition by each of the major Orders consumed by foraging godwit chicks. Invertebrates were monitored near Beluga River, AK from 2009 to 2019 along two transects according to the Arctic Shorebird Demographic Network protocol (Brown et al., 2014). Biomass was determined using taxon-specific, length-weight relationships (Rogers et al., 1977; Ganihar, 1997; Robinson et al., 2018). The 2012 and 2017 seasons (*) were short seasons and do not represent the extent of the available energy. No monitoring occurred in 2013 and 2018.

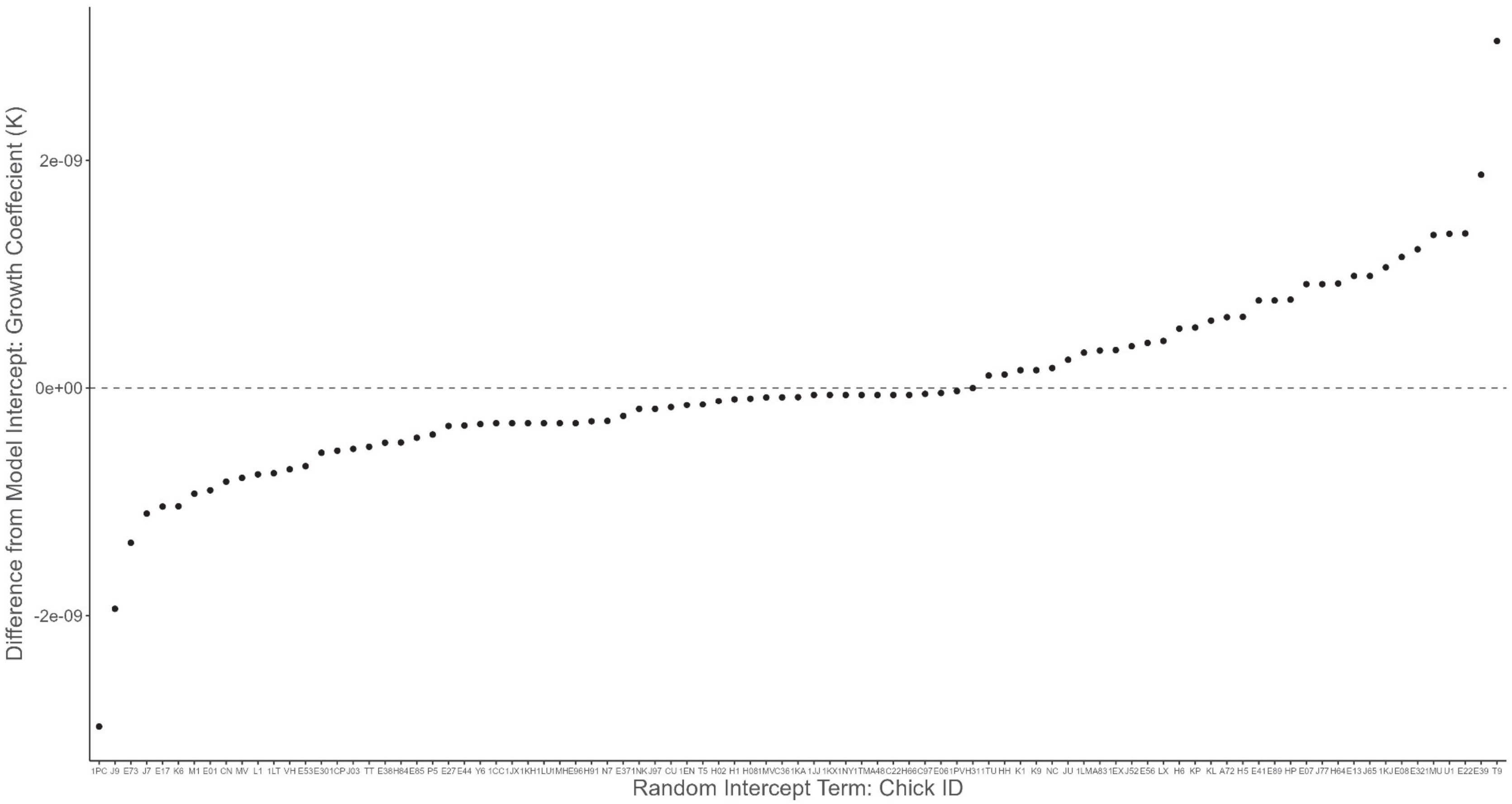

Figure S3. Non sex-specific growth curves were accounted for by allowing the intercept of the logistic growth coefficient (*K*) to vary randomly with chick ID (based on Loonstra et al., 2018). Marginal correction (y-axis) corresponds to the degree of correction per individual, which never exceeded $3{\times}10^{-9}$.

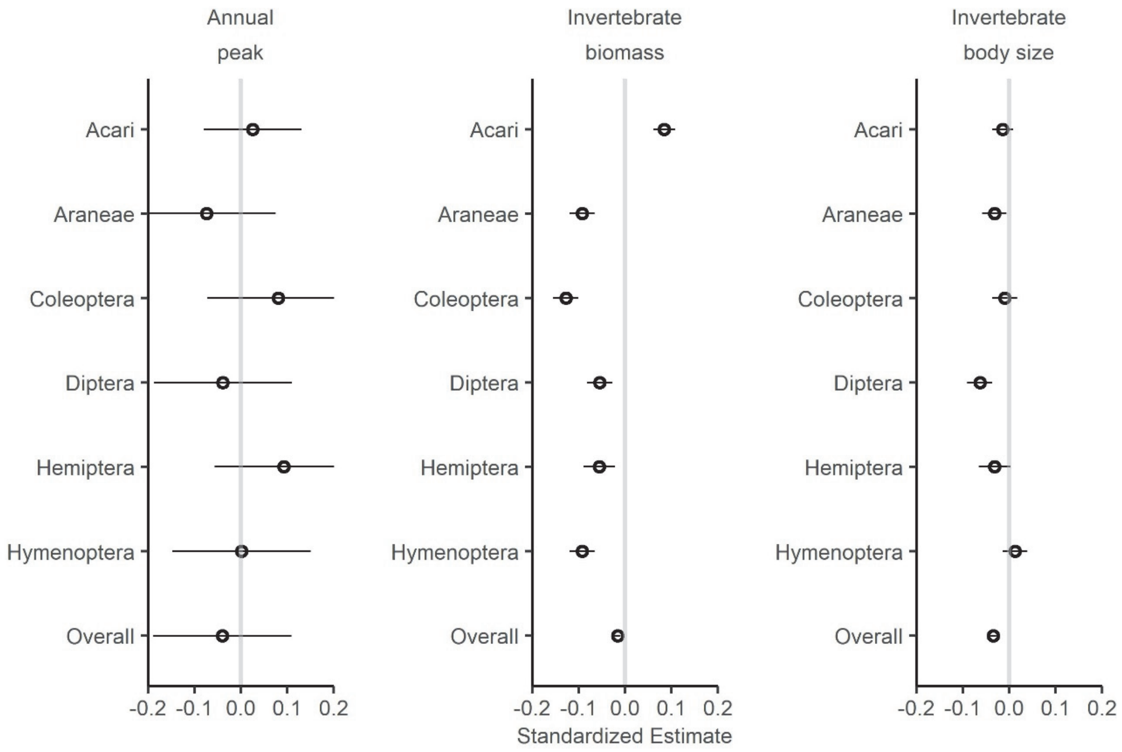

Figure S4. Interannual changes of within annual peak timing (left), daily invertebrate biomass (center), and daily median invertebrate body mass (right) of six common phylogenetic orders and the invertebrate assemblage overall. Mean effects of study year from a mixed-effect linear regression are shown as hollow circles, with 95% confidence intervals shown as horizontal lines. Orders with no consistent change oer time had intervals that crossed zero (grey line).

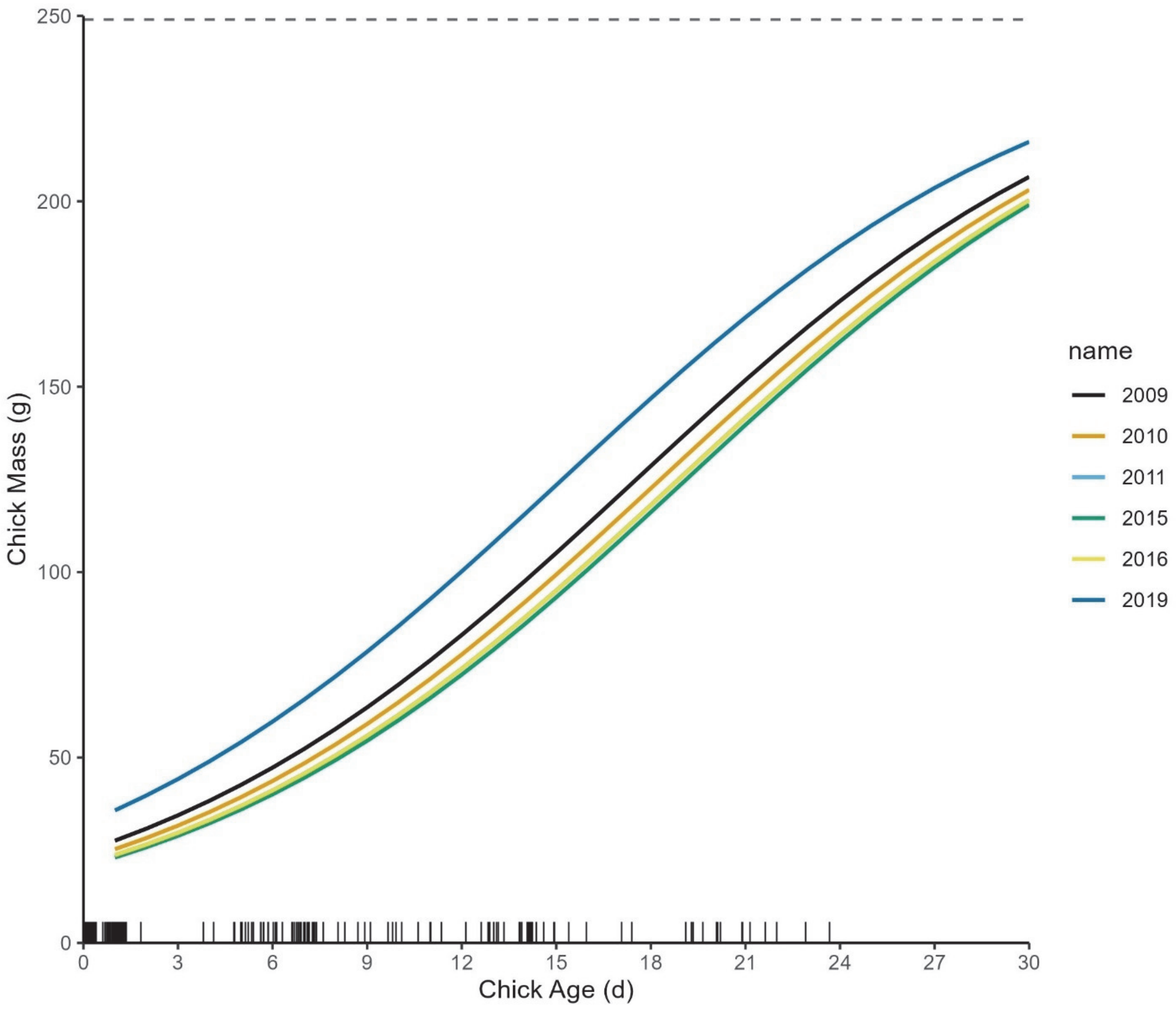

Figure S5. Annual growth curves from logistic growth equations from weekly mass-at-capture records of godwit chicks monitored near Beluga River, AK from 2009 to 2019 (excluding 2014). Assymptotic mass (249 g, Senner et al. 2017) is shown as the dotted line. Hashes on the x-axis indicate ages of the chicks sampled.

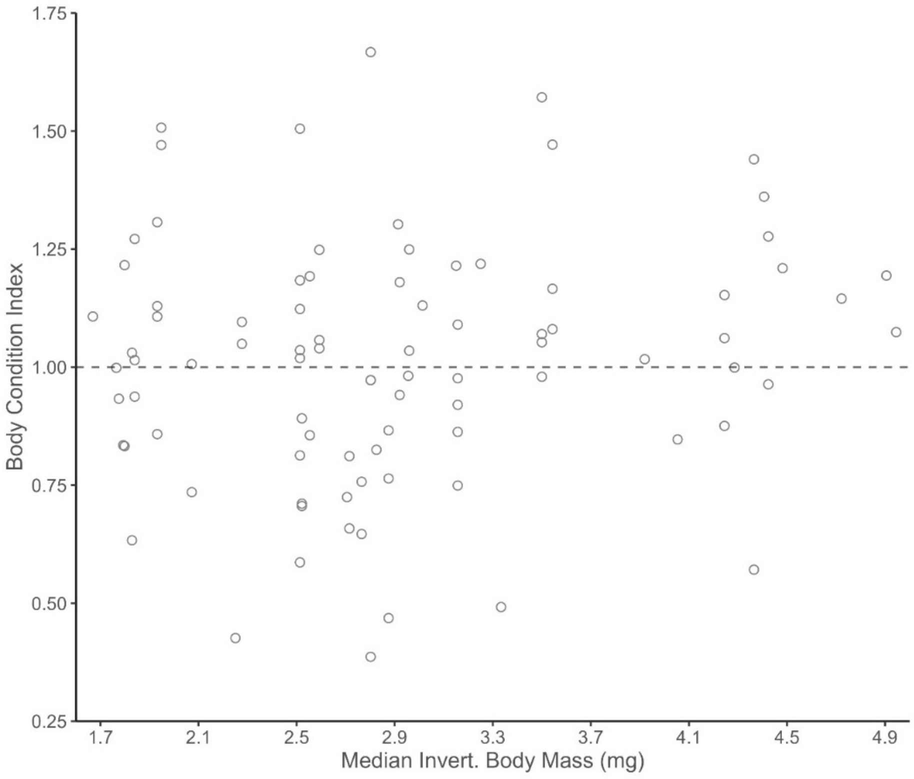

Figure S6. Correlation of daily median invertebrate body mass (mg) and godwit chick body condition index (BCI) of Hudsonian godwit chicks monitored near Beluga River, AK from 2009 to 2019. BCI (hollow points) is the ratio of the observed to expected weight gain since an individual’s last measurement. BCI>1 correspond with above average growth and BCI<1 below average growth (dashed line).

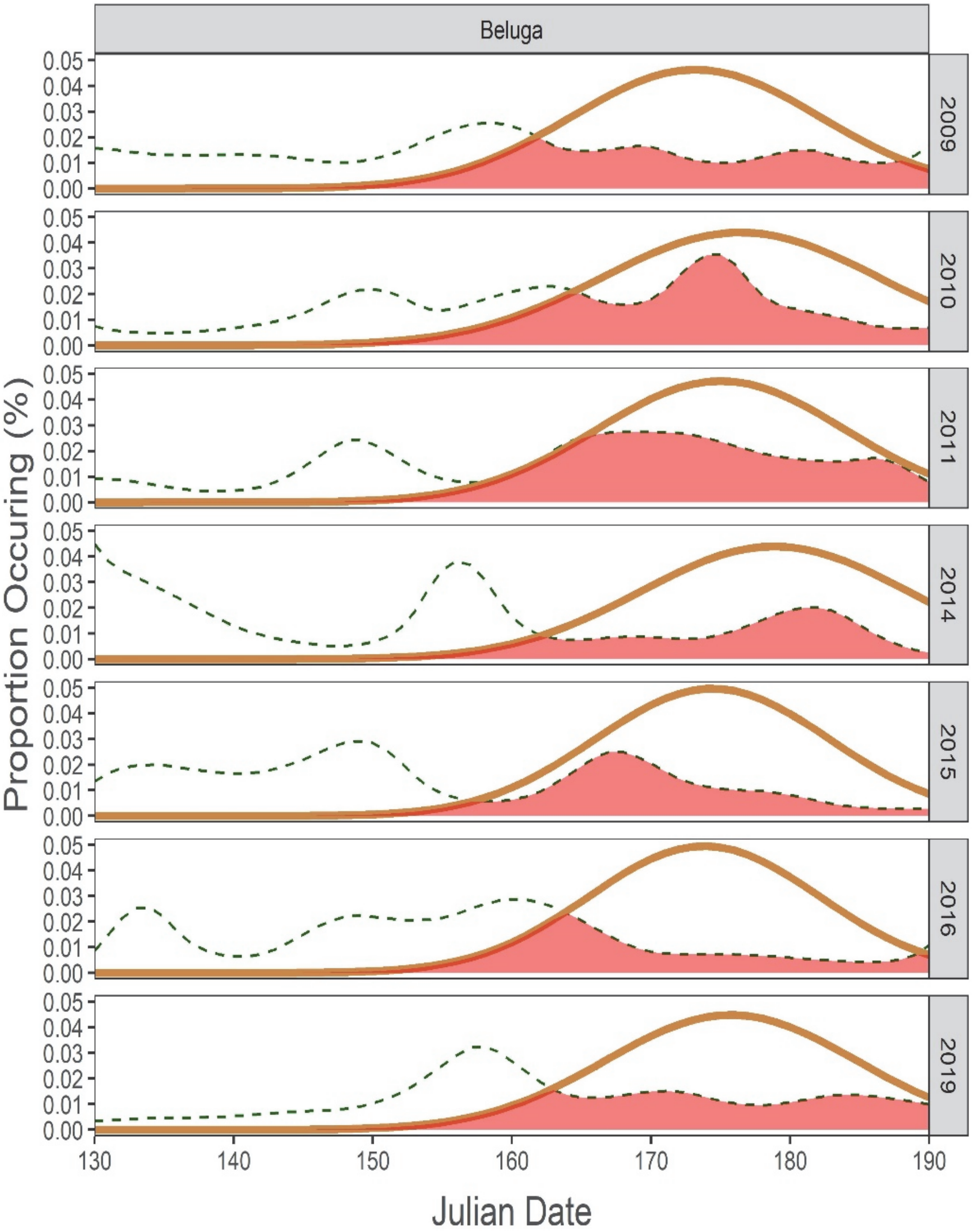

Figure S7. Overlap of the resource (i.e., daily invertebrate biomass) and Hudsonian godwit chick whole demand (i.e., age-specific energetic requirements; Kilojoules d^{-1}) curves for each year where both invertebrates and chicks were monitored near Beluga River, AK between 2009 to

2019. Demand (orange, solid line) and resource (green, dashed line) curves are represented as annual proportions, with the overlapping region (red, shaded) as a measure of ‘matching’. Each tile corresponds to a study year.

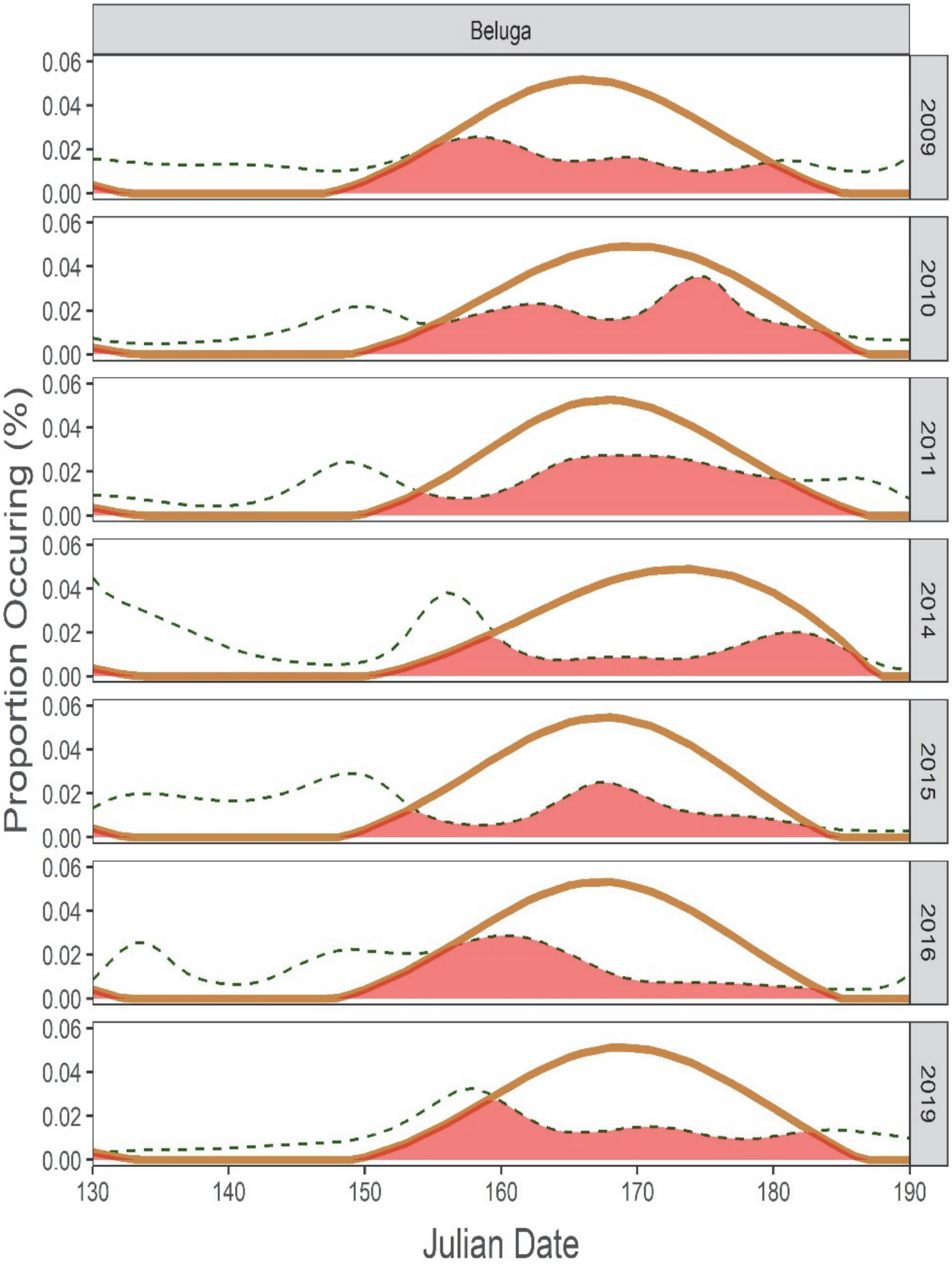

Figure S8. Overlap of the resource (i.e., daily invertebrate biomass) and Hudsonian godwit chick peak demand (i.e., number of godwit chicks at age of peak growth rate per day) curves for each

year where both invertebrates and chicks were monitored between 2009 to 2019. Demand (orange, solid line) and resource (black, dashed line) curves are represented as annual proportions, with the overlapping region (red, shaded) as a measure of 'matching'. Each tile corresponds to a study year.

References

Brown, S. C., Gates, H. R., Liebezeit, J. R., Smith, P. A., Hill, B. L., and Lanctot, R. B. (2014). Arctic Shorebird Demographics Network Breeding Camp Protocol, Version 5. *Unpubl. Paper by U.S. Fish and Wildlife Service and Manomet Center for Conservation Sciences*, 118.

Ganihar, S. R. (1997). Biomass estimates of terrestrial arthropods based on body length. *Journal of Biosciences*, *22*(2), 219–224. https://doi.org/10.1007/BF02704734

Gelman, A., & Rubin, D. B. (1992). Inference from iterative simulation using multiple sequences. *Statistical Science*, *7*(4), 457–472. https://doi.org/10.1214/ss/1177011136

Kwon, E., Weiser, E. L., Lanctot, R. B., Brown, S. C., Gates, H. R., Gilchrist, G., Kendall, S. J., Lank, D. B., Liebezeit, J. R., McKinnon, L., Nol, E., Payer, D. C., Rausch, J., Rinella, D. J., Saalfeld, S. T., Senner, N. R., Smith, P. A., Ward, D., Wisseman, R. W., & Sandercock, B. K. (2019). Geographic variation in the intensity of warming and phenological mismatch between Arctic shorebirds and invertebrates. *Ecological Monographs*, *89*(4), e01383. https://doi.org/10.1002/ecm.1383

Link, W. A., & Barker, R. J. (2006). Model weights and the foundations of multimodel inference. *Ecology*, *87*(10), 2626–2635. https://doi.org/10.1890/0012-9658(2006)87[2626:MWATFO]2.0.CO;2

Loonstra, A. H. J., Verhoeven, M. A., & Piersma, T. (2018). Sex-specific growth in chicks of the sexually dimorphic black-tailed godwit. *Ibis*, *160*(1), 89–100. https://doi.org/10.1111/ibi.12541

Maechler, M. (2020). sfsmisc: Utilities from "Seminar fuer Statistik" *ETH Zurich*. *Version 1.1-7*.

Rigby, R. A., & Stasinopoulos, D. M. (2005). Generalized additive models for location, scale and shape. *Journal of the Royal Statistical Society*, *54*, 507–554.

Robinson, S. I., McLaughlin, Ó. B., Marteinsdóttir, B., & O'Gorman, E. J. (2018). Soil temperature effects on the structure and diversity of plant and invertebrate communities in a natural warming experiment. *Journal of Animal Ecology*, *87*(3), 634–646. https://doi.org/10.1111/1365-2656.12798

Rogers, L. E., Buschbom, R. L., & Watson, C. R. (1977). Length-weight relationships of shrub-steppe invertebrates. *Annals of the Entomological Society of America*, *70*(1), 51–53. https://doi.org/10.1093/aesa/70.1.51

Sharpe, F., Bolton, M., Sheldon, R., & Ratcliffe, N. (2009). Effects of color banding, radio tagging, and repeated handling on the condition and survival of lapwing chicks and consequences for estimates of breeding productivity. *Journal of Field Ornithology*, *80*(1), 101–110. JSTOR.

Vatka, E., Orell, M., & Rytkönen, S. (2016). The relevance of food peak architecture in trophic interactions. *Global Change Biology*, *22*(4), 1585–1594. https://doi.org/10.1111/gcb.13144

Williams, J. B., Tieleman, B. I., Visser, G. H., & Ricklefs, R. E. (2007). Does growth rate determine the rate of metabolism in shorebird chicks living in the arctic? *Physiological and Biochemical Zoology*, *80*(5), 500–513. https://doi.org/10.1086/520126
